# Natural Genetic Variation and Candidate Genes for Morphological Traits in *Drosophila melanogaster*

**DOI:** 10.1371/journal.pone.0160069

**Published:** 2016-07-26

**Authors:** Valeria Paula Carreira, Julián Mensch, Esteban Hasson, Juan José Fanara

**Affiliations:** 1 Departamento de Ecología, Genética y Evolución, Facultad de Ciencias Exactas y Naturales, Universidad de Buenos Aires, Ciudad Autónoma de Buenos Aires, Argentina; 2 Instituto de Ecología, Genética y Evolución de Buenos Aires, Consejo Nacional de Investigaciones Científicas y Técnicas, Ciudad Autónoma de Buenos Aires, Argentina; Oxford Brookes University, UNITED KINGDOM

## Abstract

Body size is a complex character associated to several fitness related traits that vary within and between species as a consequence of environmental and genetic factors. Latitudinal and altitudinal clines for different morphological traits have been described in several species of *Drosophila* and previous work identified genomic regions associated with such variation in *D*. *melanogaster*. However, the genetic factors that orchestrate morphological variation have been barely studied. Here, our main objective was to investigate genetic variation for different morphological traits associated to the second chromosome in natural populations of *D*. *melanogaster* along latitudinal and altitudinal gradients in Argentina. Our results revealed weak clinal signals and a strong population effect on morphological variation. Moreover, most pairwise comparisons between populations were significant. Our study also showed important within-population genetic variation, which must be associated to the second chromosome, as the lines are otherwise genetically identical. Next, we examined the contribution of different candidate genes to natural variation for these traits. We performed quantitative complementation tests using a battery of lines bearing mutated alleles at candidate genes located in the second chromosome and six second chromosome substitution lines derived from natural populations which exhibited divergent phenotypes. Results of complementation tests revealed that natural variation at all candidate genes studied, *invected*, *Fasciclin 3*, *toucan*, *Reticulon-like1*, *jing* and *CG14478*, affects the studied characters, suggesting that they are Quantitative Trait Genes for morphological traits. Finally, the phenotypic patterns observed suggest that different alleles of each gene might contribute to natural variation for morphological traits. However, non-additive effects cannot be ruled out, as wild-derived strains differ at myriads of second chromosome loci that may interact epistatically with mutant alleles.

## Introduction

Body size is a complex character associated to fitness related traits as longevity, mate selection and tolerance to different causes of stress, among others [1–3]. Body size variation occurs across species and within species due to both environmental (i.e. nutrition, temperature, crowding, etc.) and genetic factors [4–6]. Natural variation and differences in body size related traits caused by environmental factors have been extensively studied, especially in *Drosophila* [7–14]. Further, latitudinal clines for body size traits have been described in several *Drosophila* species [15–20] and the evidence suggests that they are the product of natural selection [21–26].

Variation for morphological traits across altitudinal gradients has been less studied in *Drosophila* [27–33]. This might be due to the fact that altitudinal gradients are often thought to resemble latitudinal gradients because some environmental factors change similarly along both types of gradients. In particular, mean temperature (which decreases as latitude and altitude increase) has been considered the main environmental factor shaping both latitudinal and altitudinal clines [29, 31, 34]. However, there are environmental changes physically tied to altitude and others that are not altitude specific [35–36], and confounding both types of factors has probably impeded explaining satisfactorily the different patterns reported in separate studies [34, 37]. Several physical factors, other than temperature, that exhibit substantial change along altitudinal gradients affect insects’ performance, including air density, oxygen partial pressure and radiation [35, 37]. In this respect, flight performance, a fitness related trait in dipterans [38–40], is seriously jeopardized by the low temperature and decreased air density typical of high altitude environments [41]. This is interesting because flight performance in insects is influenced by morphological traits as body weight, thorax size, wing shape, and the composite variables wing loading (the ratio between body size and wing area) and wing aspect (the ratio between wing length and wing width) [41–43]. On one hand, studies of wing loading in natural and experimental populations of *Drosophila* [10, 18–19, 32–33, 44–47] produced evidence suggesting that low wing loading may be a flight adaptation in cold environments. On the other hand, though wing aspect has been less studied [11–12, 20, 48–49], the available evidence suggest that flies living in cold environments have longer wings.

Several studies have shown large quantities of genetic variation for morphological traits, both within and among natural populations of *D*. *melanogaster* sampled in different geographical locations [7, 16, 20, 48, 49–52] and different genomic regions have been associated with this kind of variation [21, 53–59]. In particular, some studies have shown that body size variation is mostly associated with Quantitative Trait Loci (QTL) that map on the third chromosome [21, 53–54]. In contrast, no QTLs or QTLs with minor effects on these characters were found in the other major chromosomes (i.e. second and X chromosomes). Additional studies have shown strong associations between markers within *In(3R)Payne* and size variation, suggesting that this inversion accounts for a great amount of clinal variation [60–61]. Furthermore, other studies revealed that variation at loci linked to *In(3R)Payne* significantly affects body size after correcting for the effect of the inversion itself [62–66]. More recently, Turner *et al*. [56] used a population-based re-sequencing protocol of experimentally evolved populations to map functional genetic variation for body size related traits. These authors found significant differentiation at hundreds of loci, supporting the hypothesis that the genetic architecture of body size is quite complex, involving a large fraction of the *D*. *melanogaster* genome [67]. Mapping studies also found a large number of QTL for wing shape segregating in natural populations, however their locations differed among studies [55, 57–59]. In fact, Mezey *et al*. [55] claim that these analyses have identified different QTL except for a region on the third chromosome, in which there was more agreement across studies than expected by chance. On the other hand, Palsson & Gibson [68] studied the contribution to segregating phenotypic variation of 15 candidate genes involved in wing patterning in *D*. *melanogaster* by means of the study of different wild-type alleles. Finally, independent gene specific studies found an association between sequence polymorphisms in a putative regulatory region of the *Egfr* locus (located in the second chromosome) and wing shape [69–71].

Mutagenesis by transposable elements represents a complementary approach to QTL mapping of candidate genes related to quantitative trait variation [72–73] that has been previously used to identify candidate genes for morphological traits [67, 74–75]. Both mutagenesis and QTL mapping require validation of the identified candidate loci as well as the search for natural variation (i.e. natural alleles affecting the quantitative trait). The second phase may be performed using quantitative complementation tests [72–73] which requires: i) crossing a mutant and a wild-type strain for a candidate gene to, at least, two “natural strains” and ii) measuring the trait of interest in the progeny of the four resulting genotypes [72–73]. Failure of the mutation to complement the “natural alleles” occurs when the difference between the latter is larger in the mutant background than in the wild-type background and it may be attributable to allelism or epistasis. In either case, complementation failure may be considered as an indication that the mutation uncovers natural variation for a candidate gene. Quantitative complementation test is an effective instrument to investigate the contribution of particular genes to segregating phenotypic variation [76–82], however, it has not been used to study morphological traits except wing shape [68]. This technique is even more precise when the wild type alleles of the candidate genes are in the same co-isogenic background than the mutated genes [72–73]. In this respect, chromosome substitution lines (which may be obtained by means of placing natural chromosomes extracted from wild organisms into a common genetic background) represent a powerful tool for the identification of multiple interacting loci with individually small effects influencing the studied trait [76].

In this study, our main objective was to investigate the genetic variation underlying morphological traits in natural populations of *Drosophila melanogaster* focusing our efforts on the second chromosome, the major chromosome that has been studied to a lesser extent, probably due to its apparent weaker association with morphological variation [21, 53–55, 60–62, 66]. We measured several morphological traits (face width, head width, thorax length, wing size) derived from different imaginal discs, and two composite traits (wing shape and wing loading) in 66 second chromosome substitution lines derived from flies collected in nine natural populations of *D*. *melanogaster* along latitudinal and altitudinal gradients of western Argentina. Our results revealed a strong population effect and weak clinal variation for morphological traits. As most pairwise comparisons between populations were significant, our results suggest that this factor includes information regarding latitude and altitude as well as genetic aspects that might be differentiating populations. Our analyses also showed important within-population genetic variation associated to the second chromosome, since the lines employed in our study are otherwise genetically identical. Next, we evaluated the contribution of particular genes to natural phenotypic variation, by means of quantitative complementation tests using lines bearing insertions in different candidate genes (*invected*, *Fasciclin 3*, *toucan*, *Reticulon-like1*, *jing* and *CG14478*) and second chromosome substitution lines derived from natural populations. Results of complementation tests indicated failure of complementation in all candidate genes studied and a similar genetic effect in both sexes. These results indicate that natural variation at these loci affects the studied characters, suggesting that they are Quantitative Trait Genes for the morphological traits studied. Even though the phenotypic patters observed do not allow us to discard epistasis as the genetic mechanism generating phenotypic variation among lines, they suggest that different alleles of each candidate gene contribute to variation for morphological variation observed in natural populations.

## Materials and Methods

### Establishment of substitution lines and experimental design

**Fig 1:**
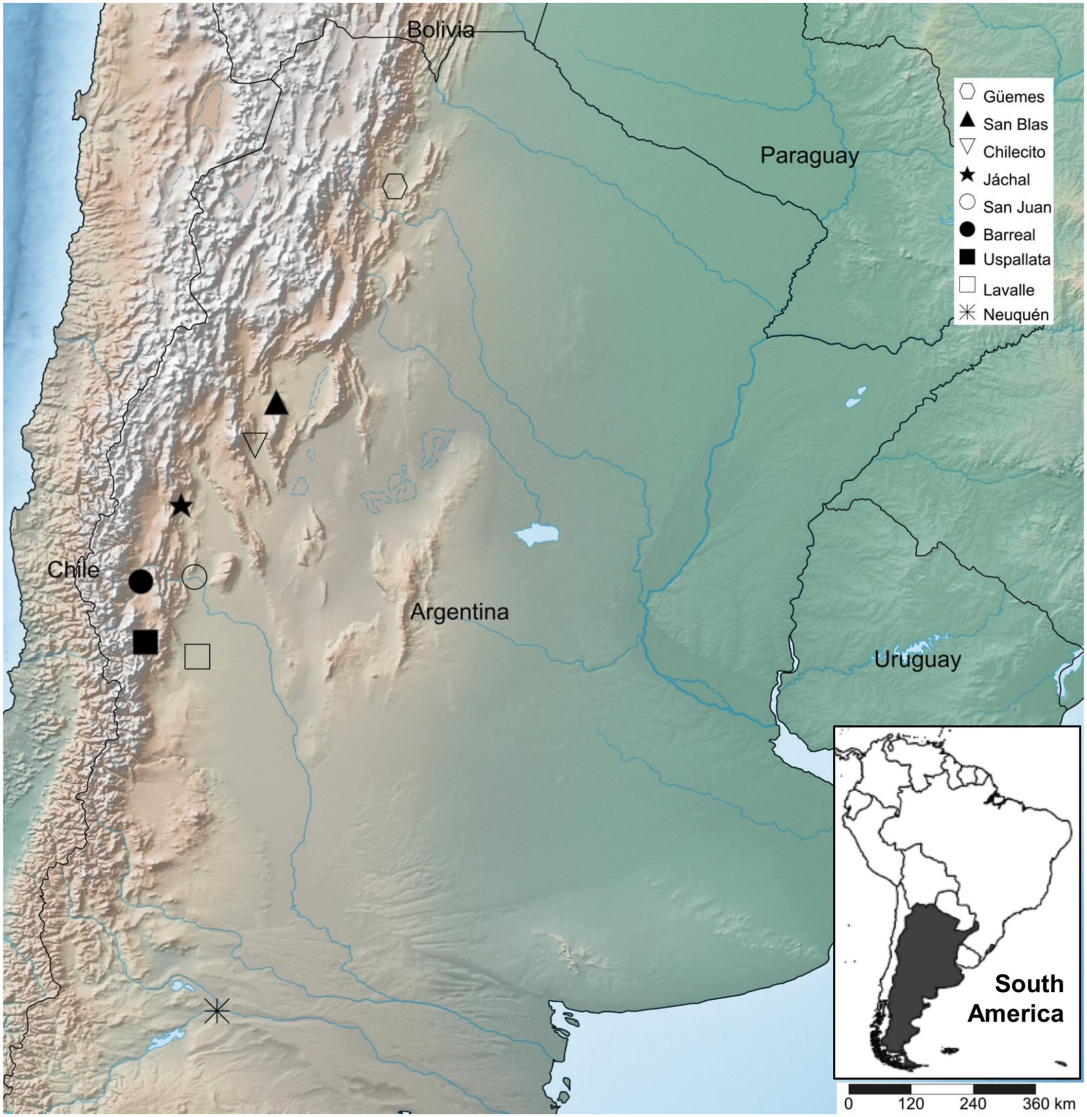
Map showing the collection sites. Localities were adults of *Drosophila melanogaster* were collected.

Adults of *Drosophila melanogaster* were collected by net sweeping on fermented banana baits in nine localities distributed along latitudinal and altitudinal gradients in Western Argentina in February 2004 and February 2005 (Fig 1, map created using SimpleMappr [83]). We did not need permission for collecting flies at these sites as *D*. *melanogaster* is a human commensal and a cosmopolitan non endangered species (https://cites.org/). Captured flies were sexed in the laboratory and isofemale lines were set up by rearing the progeny of single females [84]. All lines were maintained by full-sib mating for 10 generations on cornmeal-molasses-agar medium under standard conditions (25±1°C and 60–70% of humidity with a 12:12 light:dark photoperiod). Next, a single second chromosome was extracted from each isofemale line and substituted into the genetic background of an isogenic *Canton-S* B (hereafter *IsoB*) strain by standard techniques using balancer chromosomes (S1 Fig). To construct second chromosome substitution lines, one male of each isofemale line was crossed to [*w*; *Cy*/IsoB; *Sb*/IsoB] females [generation 0 (G0)]. A single [*w*; *Cy*/+; *Sb*/+] male from the progeny of each cross was crossed to [*w*; *Cy*/*Sp*; IsoB] females (G1). Then, a single [*w*; *Cy* or *Sp*/+; *Sb*/IsoB] male was crossed to [*w*; *Cy*/*Sp*; IsoB] females (G2). Subsequently, [*w*; *Cy* or *Sp*/+; IsoB] females and males were crossed eliminating the *Sb* balancer (G3). Finally, females and males of genotype [*w*; +; IsoB] were crossed and the *Cy* and *Sp* balancers eliminated to maintain the second chromosome substitution line originated (G4). As a result, we generated 66 second chromosome substitution lines, each one isogenic for one wild derived second chromosome in an isogenic background common to all lines. The number of lines per sampling locality along with geographical coordinates and altitude are given in S1 Table.

The obtaining of adult flies for morphological quantification was as follows. For each substitution line, 300 pairs of sexually mature flies were placed in oviposition chambers for egg collection during 8 hours. Eggs were allowed to hatch and batches of 30 first-instar larvae were transferred to culture vials containing a standard cornmeal-agar-molasses medium (4 replicates per line). Larvae were raised at 25±1°C and 60–70% humidity with a 12:12 light:dark photoperiod until adult emergence. All adults emerged from each vial were stored at -20°C until quantification of morphological traits was performed.

### Morphological data acquisition

Five flies of each sex were randomly chosen from each vial (20 males and 20 females per line) and the head, the thorax and the wings of each individual were removed and placed on a slide keeping their relative position. Separate images for 3-D structures (i.e., head and thorax) and flat structures (i.e., wings) were captured using a binocular microscope (10x) with an attached digital camera connected to a computer. Different morphological traits were measured using tpsDig [85], exactly as in previous works [67, 74–75]. Face width (FW, the smallest distance between the eyes), head width (HW, the distance between the right and the left side of the head capsule), and thorax length (TL, the distance between the anterior margin of the thorax and the tip of the scutellum) were estimated directly from the pictures (S2 Fig). For the estimation of wing traits, 11 landmarks were digitized on the ventral face of the left wing of each fly (S3 Fig). A single wing size (WSi) measure, centroid size, was calculated by taking the square root of the sum of squared distances between each landmark and the centroid (the point whose coordinates are the means of the x and y coordinates of all landmarks) of each wing. Wing shape (WSh) was studied using the Procrustes generalized least squares procedure [86], which allows for the superimposition of all the wings. This procedure eliminates variation in wing size, position and orientation and allows for the examination of differences in position of landmarks. Then, we estimated the Procrustes distance (PD) to the reference of the sample (the mean wing shape corresponding to the flies included in the respective analysis) using tpsSmall [87]. WSi and WSh were log-transformed prior to all analyses. Finally, we estimated wing loading (WL) as the ratio between TL and Log_10_WSi.

### Quantitative complementation tests

To examine the contribution of genes known to affect morphological traits [67, 74–75] to natural variation for these traits we performed quantitative complementation tests. First, we selected six mutant lines known to differ from the control line for several morphological traits, in which the affected genes (candidate genes) are located in the second chromosome [67, 75]. These lines (acquired from the Bloomington *Drosophila* Stock Center, http://flystocks.bio.indiana.edu/) were obtained using a gene-trap system which allows recovering only fly lines whose genes are inactivated by a *P*-element insertion [88] and belong to a large collection of independent homozygous viable single *P[GT1]*-element insertion lines, constructed in a co-isogenic *Canton-S* background as part of the Berkeley *Drosophila* Genome Project [88–89]. The mutant lines selected for the present study are BG00846, BG02023, BG02065, BG02081, BG02314 and BG02690, each carrying a mutation affecting the candidate genes *invected* (*inv*), *Fasciclin 3* (*Fas3*), *toucan* (*toc*), *Reticulon-like1*, *jing* and *CG14478* respectively [67, 74–75]. These mutations showed different phenotypic effects on the morphological traits studied (S2 Table) and they were selected after considering the diversity of the effects, the genomic location of the mutation and the availability of stocks. From the set of second chromosome substitution lines we selected six lines derived from different populations that exhibited divergent phenotypes for several body size related traits in both sexes, to increase the chances of detecting natural variation for the studied candidate genes (i.e. different alleles). Selected substitution lines are Güemes 269, San Blas 29 and Neuquén 58, among the strains which showed larger sizes, and Chilecito 29, Jáchal 5 and Lavalle12, among those which showed smaller sizes (see below).

Substitution lines were crossed individually with flies of the *P*-element insertion lines carrying the mutated alleles of one of the six candidate genes and, in parallel, with a *P*-element free insertion line (IsoB) with the same genetic background (*Canton-S* B) used as control line). The F1 progenies of these crosses were m/+i and IsoB/+i, respectively, where m is the mutant allele (derived from the *P*-element insertion line) and +i represents the wild derived allele of the respective candidate gene. To obtain F1s, virgin females of each substitution line were crossed with males of the mutant or IsoB lines in egg collecting chambers and batches of 30 F1 first-instar larvae were transferred to culture vials with fresh medium. Morphological traits were measured as explained before in five flies of each sex from each vial making a total of 20 males and 20 females per cross and a grand total of ~2800 flies.

### Statistical analyses

Statistical analyses were performed for each morphological character separately because correlation analyses for pairs of characters (performed using the mean value corresponding to each substitution line) revealed that traits are not highly correlated (S3 Table). Similar results were obtained in a previous work [67] that allowed us to identify candidate genes for body size related traits, some of which were further studied in the present investigation. This agreement reinforced the idea of analyzing each trait separately.

To analyze the effects of latitude, altitude and population on morphological traits, we studied the fit of models incorporating each one of these factors independently as well as latitude and altitude simultaneously. As these statistical analyses were done in R [90], tested models may be represented as follows: Model 1: lm (X~Latitude); Model 2: lm (X~Altitude); Model 3: lm (X~Latitude*Altitude) and Model 4: lm (X~Population); where X represents the character. Sexes were analyzed separately because the large sexual dimorphism observed for the studied traits in *D*. *melanogaster* [67, 74–75] may confuse the relative minor effect of the factors under study (i.e., latitude, altitude and population). Then, we compared the fit of the models for each combination of sex and morphological trait using the Akaike Information Criterion (AIC), with the package AICcmodavg [91]. Finally, we performed Tukey tests to compare the mean value of each character between populations, for males and females separately. An example of the scripts used is shown in S1 Appendix.

Next, two way ANOVAs were conducted to investigate sources of variation within populations for each trait separately, according to the model: Y = μ + L + S + L x S + Є; where μ is the overall trait mean, L is the random line effect, S is the fixed effect of sex, and Є represents the error. Variance components for random sources of variation (L and L x S) were estimated in each case. These analyses were only performed for populations in which sample sizes were of at least five second chromosome substitution lines and the mixed model was fit by expected mean squares.

Data derived from crosses involving all substitution lines (one cross with the mutant line and the other with IsoB) were analyzed separately for each candidate gene. Each morphological trait was analyzed with an ANOVA according to the model: Y = μ + G + L + S + G x L + G x S + L x S + G x L x S + Є; where μ is the overall mean of the trait, G, L and S are the fixed effects of Genotype (mutant or IsoB allele), second chromosome substitution Line and Sex, respectively, and Є is the error.

The criteria that warrant quantitative failure of complementation are either a significant L x G interaction or L x G x S interaction [73, 92]. Because substitution lines varied at multiple loci in the second chromosome, epistasis between these other genes derived from natural populations and the IsoB allele for the studied gene may contribute to phenotypic variation [73, 92–93]. In the ideal scenario, significant differences would be detected only between wild type alleles in the mutant background as wild type alleles in the control background would be masked by the dominant IsoB allele. These differences have been traditionally studied through post-hoc contrasts as the experimental designs have normally included just a few wild type alleles each time [76–80]. However, such analysis may become cumbersome when several wild type alleles are tested simultaneously. In this sense, one way to assess if quantitative failure of complementation may be explained by the presence of different alleles at the candidate locus studied, is to verify that variance among lines in the IsoB background is not greater than variance among lines in the mutant background [81, 94]. Thus, we used an *F*-statistic to examine the equality of genetic variances (Line and Line x Sex variances) corresponding to the IsoB and mutant backgrounds [94]. If this criterion is met, the existence of different alleles for a candidate gene affecting natural variation of a certain trait cannot be discarded.

All statistical analyses were performed using the STATISTICA software package [95] except otherwise stated. Bonferroni correction for multiple tests was applied whenever results from multiple tests were combined in one final conclusion.

## Results

### Inter- and intra-population variation for morphological traits

The effects of latitude, altitude and population on morphological traits were investigated testing the fit of models incorporating each factor independently as well as latitude and altitude simultaneously. These analyses showed a highly significant effect of latitude on all traits except WSh in both sexes and a significant effect of altitude on HW, TL, WL and WSi in females and all traits except FW in males (S4 Table, S4 Fig). In particular, all body size related traits (FW, HW, TL, WL and WSi) increased with latitude in both sexes and decreased with altitude except for FW, which was not significant in both sexes (S4 Table, S4 Fig). The model incorporating the interaction between latitude and altitude was only significant for FW, HW and WSh in females and for HW and WSh in males (S4 Table). According to slope estimates, in general, latitude had the largest effect on trait variation (S4 Table). Visualization of wing deformations showed that some landmarks exhibited larger displacements than others with latitude and the magnitude and direction of these changes was very similar across sexes (S5 Fig). Besides, the posterior crossvein showed a similar movement in both sexes: an inclining towards the proximal part of the wing as latitude increased (S5 Fig). In general, we found more elongated wings at higher latitudes and more rounded wings at lower latitudes (S5 Fig).

Finally, the effect of population was highly significant for all traits in both sexes (Table 1). In fact, the comparative analyses showed that the model incorporating only the population factor was always the best (i.e., it showed the lowest AIC value) in comparison with the other three models, generally followed by the model incorporating latitude and altitude simultaneously (S5 Table). Finally, Tukey tests revealed that all the populations differed significantly from the others at least for one trait in one sex except for San Juan, which did not show significant differences with respect to Barreal and Lavalle for any character (S6 Table). These comparisons showed that Neuquén was the most divergent population (76 significant contrasts out of 96 performed tests, 79%), followed by Jáchal (68% of significant contrasts) and Uspallata (54% of significant contrasts), while the population showing less significant contrasts was San Juan (24% of significant contrasts; S6 Table).

**Table 1:** Model testing the population effect on morphological traits.

	Females						Males				
	SS	df	*F*	p-value	MRS		SS	df	*F*	p-value	MRS
						**Face Width**					
**Population**	7800	8	12.93	<2.2e-16	0.08	**(FW)**	8646	8	11.12	3.09e-15	0.07
**Residuals**	87449	1160					112528	1158			
						**Head Width**					
**Population**	26345	8	24.38	<2.2e-16	0.14	**(HW)**	22912	8	22.13	<2.2e-16	0.13
**Residuals**	156675	1160					149863	1158			
						**Thorax Length**					
**Population**	36632	8	21.04	<2.2e-16	0.13	**(TL)**	36193	8	23.26	<2.2e-16	0.14
**Residuals**	252688	1161					225475	1159			
						**Wing Loading**					
**Population**	109.76	8	18.09	<2.2e-16	0.11	**(WL)**	115.19	8	20.96	<2.2e-16	0.13
**Residuals**	872.96	1151					789.35	1149			
						**Wing Size**					
**Population**	0.05	8	17.16	<2.2e-16	0.11	**(WSi)**	0.03	8	11.34	1.47e-15	0.07
**Residuals**	0.42	1157					0.39	1153			
						**Wing Shape**					
**Population**	1.22	8	11.57	6.56e-16	0.07	**(WSh)**	1.31	8	11.89	<2.2e-16	0.08
**Residuals**	15.19	1157					15.85	1154			

Principal results of the best model (i.e., the model with the lowest AIC value), Model 4: lm (X~Population), where X represents the character (see Materials and Methods for more details). SS: Sum of Squares, df: degrees of freedom, MRS: Multiple R-squared.

The ANOVAs performed for each population and trait revealed that sexes were highly different for all body size traits (Table 2). They also showed that the line factor was significant in most cases (exceptions: FW in Güemes and Lavalle, HW in Neuquén and WSh in all populations; Table 2). However, the line by sex interaction was significant for most of the cases with non-significant line and/or sex effects (HW in Neuquén and WSh in all populations; Table 2). In most populations the genetic components (L and L x S) accounted from 6 to 41% of overall phenotypic variance indicating significant within-population genetic variation, which must be due to the second chromosome as the lines are otherwise genetically identical (Table 2).

**Table 2:** Within-population quantitative genetic analyses for morphological traits.

		Güemes	San Blas	Chilecito	Uspallata	Lavalle	Neuquén
**Face Width**	L	2.67 (6)	5.97* (13)	5.91** (22)	7.93* (10)	3.35 (10)	3.85* (12)
**(FW)**	S	53.28***	91.56***	65.91***	110.28***	45.45***	64.61***
	L x S	1.47 (2)	1.03	1.87* (4)	0.62 (0)	1.64 (3)	1.56 (3)
**Head Width**	L	9.90** (21)	7.40** (16)	10.91*** (29)	12.48** (22)	18.32*** (22)	1.84 (5)
**(HW)**	S	259.45***	166.36***	148.13***	346.61***	261.09***	103.17***
	L x S	1.07 (0)	1.03 (0)	1.32 (1)	0.93 (0)	0.58 (0)	2.13* (6)
**Thorax Length**	L	26.92*** (41)	11.37** (21)	12.87*** (28)	6.63* (12)	17.47*** (17)	4.32* (11)
**(TL)**	S	635.04***	445.83***	553.01***	614.29***	753.86***	373.16***
	L x S	0.98 (0)	0.88 (0)	1.03 (0)	0.88 (0)	0.42 (0)	1.18 (1)
**Wing Size**	L	24.32*** (29)	19.49*** (19)	12.58*** (31)	7.05* (14)	12.26** (26)	13.05*** (30)
**(WSi)**	S	1144.91***	851.17***	483.35***	1002.26***	612.01***	1113.35***
	L x S	0.66 (0)	0.44 (0)	1.25 (1)	1.01 (0)	1.05 (0)	1.15 (1)
**Wing Loading**	L	26.64*** (40)	14.10*** (21)	11.07*** (28)	6.35* (11)	14.56** (17)	4.10* (10)
**(WL)**	S	616.24***	508.64***	492.62***	581.50***	601.22***	347.26***
	L x S	0.97 (0)	0.69 (0)	1.18 (1)	0.87 (0)	0.50 (0)	1.11 (1)
**Wing Shape**	L	2.20 (8)	3.30 (14)	1.90 (14)	0.01 (0)	2.00 (10)	1.70 (11)
**(WSh)**	S	0.30	0.40	0.01	0.01	0.90	0.60
	L x S	2.80**(8)	2.80** (8)	8.10*** (27)	3.00* (8)	4.40*** (15)	7.50*** (26)

An ANOVA was performed with data of each population and trait, following a model with line (L) and sex (S) as factors. *F*-values corresponding to all sources of variation are shown. Percentage of total phenotypic variance explained by each random source of variation (L and L x S) is shown between parentheses.

*p<0.05,

**p<0.01,

***p<0.001.

### Natural variation in candidate genes for morphological traits

Second chromosome substitution lines were ordered according to mean value independently for each body size trait (from the minimum to the maximum value). Then, a score from 1 to 66 was assigned to each line according to its position in the ranking corresponding to each trait. Finally, the four scores (one per each body size trait: FW, HW, TL and WSi) were added up and a final ranking of the 66 lines was obtained using the composite scores. No line presented the minimum or the maximum possible value (4 and 264 respectively) in either sex (S6 Fig); indicating that no line showed the smallest or the largest mean value for all traits in males or females. In this respect, correlation analyses between the scores representing body size traits revealed that they are not highly correlated (S7 Table), as expected from the correlation analyses using the raw data of morphological variables (S3 Table). Therefore, we decided to analyze different morphological traits separately in the following statistical analyses. In general, the six second chromosome substitution lines selected for the complementation tests exhibited extreme phenotypes for several body size related traits in both sexes and showed relatively large (Güemes 269, Neuquén 58 and San Blas 29) or small (Chilecito 29, Jáchal 5 and Lavalle 12) composite scores in both sexes (S6 Fig).

The ANOVAs performed with the data of the descendants of the crosses between the substitution lines and each mutant line (plus the values corresponding to the crosses between the same substitution lines and the IsoB line as a control) showed that the line by genotype interaction was significant in most cases, being FW and WSh the traits that showed less significant results for this interaction (Table 3). In general, our results suggest failure of complementation for all six candidate genes, at least for a couple of traits in each case (Table 3). In contrast, the triple interaction (L x G x S) was significant only in three cases (WSh in *Reticulon-like1* and *jing* and TL in *CG14478*) implying that, in general, genetic variation affects both sexes similarly (Table 3).

**Table 3:** Genetic complementation tests for morphological traits.

	FW	HW	TL	WSi	WSh	FW	HW	TL	WSi	WSh
	***invected***					***Fasciclin 3***				
**L**	14.36***	13.17***	4.42***	10.54***	5.88***	4.57***	7.39***	3.06*	13.81***	2.38*†
**G**	19.90***	17.39***	18.16***	8.49**	0.53**	2.45	0.82	10.84**	0.04	0.16
**S**	254.36***	477.02***	861.30***	1275.43***	0.15	144.19***	326.32***	568.38***	1200.76***	0.01
**L x G**	1.42	9.32***	9.01***	5.84***	1.59	5.40***	5.84***	5.49***	3.34**	1.69
**L x S**	0.91	0.54	1.90	1.06	10.73***	1.20	1.77	1.44	1.38	5.90***
**G x S**	10.42**	0.82	2.74	0.15	23.87***	0.69	4.46	1.14	1.82	0.01
**L x G x S**	0.79	0.76	0.78	0.50	2.34*†	0.88	1.12	2.08	0.62	2.06
	***Reticulon-like1***					***jing***				
**L**	9.05***	7.09***	2.06	3.92**	5.10***	13.92***	15.28***	11.03***	16.60***	2.50*†
**G**	0.10	19.13***	11.97***	3.39	0.84	1.80	0.08	2.76	1.36	1.39
**S**	166.58***	522.86***	775.09***	1283.84***	0.41**	196.34***	532.89***	726.40***	1321.21***	0.61
**L x G**	4.24***	10.48***	11.42***	9.02***	1.07	1.96	5.87***	1.69	3.42**	4.48**
**L x S**	1.20	1.25	0.79	0.42	5.15***	0.28	1.72	0.49	0.98	3.16**
**G x S**	0.41	1.52	2.50	0.23	4.81*†	2.34	1.53	0.01	0.00	0.01
**L x G x S**	1.32	0.94	1.56	1.49	3.42**	0.26	1.57	1.96	1.57	3.83**
	***toucan***					***CG14478***				
**L**	11.51***	9.24***	7.61***	9.55***	1.88	9.73***	11.66***	4.80***	12.14***	3.13**
**G**	0.17	2.66	0.03	0.19	5.44*	2.11	4.33*†	4.66*	5.30*	0.26
**S**	156.34***	405.48***	646.40***	1084.18***	9.66**	159.45***	351.04***	643.52***	1097.69***	0.24
**L x G**	1.73	4.07**	3.66**	2.49*†	1.45	1.76	5.77***	7.87***	2.82*	1.92
**L x S**	1.22	2.91*	1.37	0.87	4.52***	0.93	1.83	0.65	1.87	9.78***
**G x S**	0.11	0.03	0.00	1.54	3.24	0.55	0.08	0.00	0.43	13.34***
**L x G x S**	1.40	1.31	0.92	1.05	1.70	0.77	1.14	2.61*	0.83	2.15

ANOVAs performed with the values corresponding to individuals derived from crosses between six substitution lines and a mutant line (plus the values corresponding to the crosses between the same substitution lines and a control line). L, G and S are the effects of Substitution Line, Genotype (mutant or control) and Sex, respectively. *F*-values corresponding to all sources of variation are shown. *invected*, *Fasciclin 3*, *toucan*, *Reticulon-like1*, *jing* and *CG14478* are the candidate genes affected by the *P*-element insertion in the mutant lines used. FW: Face Width, HW: Head Width, TL: Thorax Length, WSi: Wing Size.

*p<0.05,

**p<0.01;

***p<0.001.

^†^ Not significant after Bonferroni correction for multiple tests (P_B_ = 0.025).

All comparisons between genetic variances (Line and Line x Sex) over mutant and IsoB (control) backgrounds yielded non-significant results for all candidate genes (Table 4) indicating that variances were homogeneous. A greater variance over the mutant background with respect to the control background would have suggested allelism as the predominant genetic mechanism generating phenotypic variation among lines. On the other hand, a greater variance over the control background in comparison to the mutant background would have indicated epistasis.

**Table 4:** Comparison between phenotypic variances over mutant and control backgrounds.

	Face width		Head width		Thorax length		Wing size		Wing shape		
	σ_c_ = 13.70		σ_c_ = 24.53		σ_c_ = 29.84		σ_c_ = 0.58x10^-4^		PD (x10^-3^) §		
	σ_m_	*F*	σ_m_	*F*	σ_m_	*F*	σ_m_ (x10^-4^)	*F*	σ_m_ (x10^-3^)	σ_c_	*F*
***invected***	11.64	1.18	20.53	1.20	14.04	2.13	0.38	1.53	6.09	2.17	0.36
***Fasciclin 3***	3.22	4.25	4.38	5.60*	9.60	3.11	0.37	1.57	1.98	2.16	1.09
***toucan***	7.32	1.87	11.14	2.20	9.27	3.22	0.11	5.27*	1.24	2.11	1.70
***Reticulon-like1***	9.54	1.44	6.81	3.60	16.65	1.79	0.14	4.14	2.48	2.31	0.93
***jing***	11.32	1.21	13.96	1.76	12.76	2.34	0.52	1.12	1.86	2.30	1.24
***CG14478***	3.82	3.59	14.72	1.67	17.91	1.67	0.32	1.81	5.62	2.17	0.39

Variances over mutant (σ_m_) and control (σ_c_) backgrounds for each morphological trait. *F* values were compared to F_5,5_ = 5.05.

*p = 0.041 (not significant after Bonferroni correction for multiple tests, P_B_ = 0.025).

^§^ PD is a measure of the difference between the shape of an individual and the mean shape of the sample to which that individual belongs. Therefore, PD values depend on the individuals composing the studied sample. In quantitative complementation tests, data corresponding to each candidate gene are analyzed separately. Each analysis includes the phenotypic values of the descendants of the crosses between the substitution lines and the respective mutant line. It also includes the values corresponding to the crosses between the same substitution lines and the control line. Even though the control individuals included in different analyses are the same, PDs of these individuals change from one analysis to the other because the sample contains different mutant individuals in each case. Therefore, the mean wing shape of the sample changes from one analysis to the other and, consequently, PD values of the control individuals also change. This matter only concerns PDs which are relative values, as the other traits are absolute measurements which do not change with the sample.

**Fig 2:**
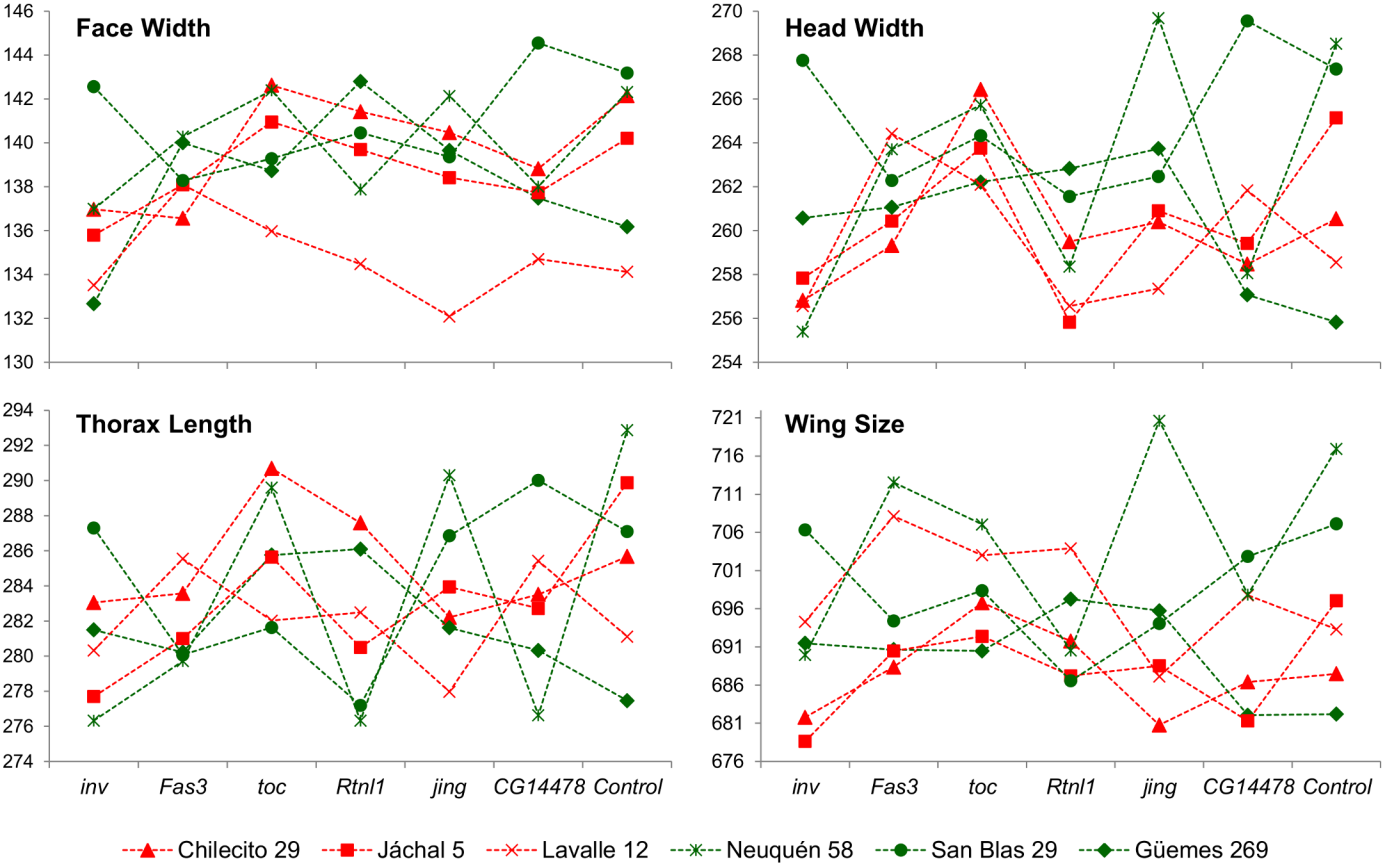
Quantitative Complementation Tests for body size related traits. Mean values of body size traits (Face Width, Head Width, Thorax Length and Wing Size) corresponding to individuals derived from complementation tests between second chromosome substitution lines (Chilecito 29, Jáchal 5, Lavalle 12, Neuquén 58, San Blas 29 and Güemes 269) and the laboratory lines (mutant and control lines) averaged across sexes. Crosses with substitution lines which had previously shown larger and smaller sizes are indicated in green and red respectively. *inv* (*invected*), *Fas 3* (*Fasciclin 3*), *toc* (*toucan*), *Rtnl1* (*Reticulon-like1*), *jing* and *CG14478* are the candidate genes affected by the *P*-element insertion in the mutant strains used. The control is a *P*-element free insertion line with the same genetic background than the rest of the strains used.

Fig 2 shows the mean value of each body size trait (FW, HW, TL and WSi) corresponding to individuals derived from crosses between the wild-derived lines (second chromosome substitution lines) and the laboratory lines (mutant and control lines) averaged across sexes. In general, it shows that the control allele is not always dominant over the natural alleles (i.e., differences between means corresponding to the descendants of crosses between the wild-derived lines and the control lines may be observed for all traits). It also reveals differences between means corresponding to the descendants of crosses between the wild-derived lines and the mutant lines for all traits. However, the complementation effect seems to be strongly dependent on the candidate gene studied as well as the substitution line (i.e., the second chromosome) analyzed. In this sense, “a larger genetic background” (i.e., substitution lines which had previously shown larger sizes) did not necessarily yield larger descendants carrying one copy of the natural allele of the respective gene. The same seems to be true for “smaller genetic backgrounds” (i.e., substitution lines which had previously shown smaller sizes). For example, different outcomes may be appreciated when phenotypic means corresponding to descendants of the substitution lines San Blas 29 (one of the lines which had previously shown a larger size) and Chilecito 29 (one of the lines which had previously shown a smaller size) are inspected (Fig 2). Differences between means are larger over the mutant background with respect to the control background when FW and TL are analyzed in descendants of *CG14478* and *toucan* mutants. Even though both of these constitute examples of the expected pattern for allelism, only the effects observed in the first one (FW and TL in descendants of *CG14478*) are in the right direction, as FW and TL means corresponding to descendants of San Blas 29 are larger than those of Chilecito 29 (Fig 2). On the contrary, differences between means are smaller over the mutant background with respect to the control background when HW and WSi are analyzed in descendants of *Reticulon-like1* and *toucan* mutants, which constitute examples of the expected pattern for epistasis (Fig 2). Therefore, different patterns may be easily observed for different traits and candidate genes suggesting that both, allelism and epistasis, may be generating the observed phenotypic variation among lines.

## Discussion

We studied the genetic component of variation of several morphological traits (face width, head width, thorax length, wing size, wing shape and wing loading) in 66 second chromosome substitution lines derived from nine populations of *D*. *melanogaster* sampled along latitudinal and altitudinal gradients in western Argentina. Our results showed clinal variation for most morphological traits in both sexes, which must be due to genetic variants located in the second chromosome, as the studied lines are otherwise genetically identical. However, morphological variation seems to be better explained by intrinsic population properties. Furthermore, our study revealed differences among most populations and within-population genetic variation for all characters that may be explained by allelic variation in genes that map in the second chromosome acting either additively and/or interacting with other regions of the genome. We also examined the contribution of six candidate genes (*invected*, *Fasciclin 3*, *toucan*, *Reticulon-like1*, *jing* and *CG14478*) located in the second chromosome [67, 74–75] to natural variation for morphological traits by means of quantitative complementation tests between mutant lines for each one of the candidate genes and six second chromosome substitution lines. Our results suggest failure of complementation for all candidate genes which seems to affect size traits similarly in both sexes. Even though the results of complementation tests do not rule out epistasis, they suggest that different alleles of each candidate gene contribute to morphological variation in natural populations.

### Weak clinal variation and strong differences among populations for morphological traits

Our analyses testing the effects of latitude on morphological traits indicate that all body size related traits increase with latitude in both sexes, in agreement with surveys in other regions around the world [7, 20, 25, 29, 34, 96]. Despite the use of different body size estimators this clinal pattern has been found in all continents so far. Other works assessed wing size traits as wing area [23–24, 34, 54] and wing length [51, 97] and some of them attempted to link morphological clines to inversions segregating in natural populations and molecular markers within them [98–99]. In this vein, latitudinal clines in inversion frequencies have been shown for the cosmopolitan *In(2L)t* [100–102]. Thus, it may be argued that the clines in morphological traits described in the present study can be related to inversion clines. Even though we detected the presence of four inversions in our sample (i.e., *In(2L)t* in one line of Jáchal, one line of Neuquén and one line of Uspallata; and *In(2R)NS* in one line of Neuquén), most of the lines used in our study carry the second chromosome standard arrangement, ruling out *In(2L)t* as a possible explanation for the latitudinal cline for morphological traits. These results agree with studies showing that body size variation is not correlated with *In(2L)t* frequency in populations of the western coast of South America [20]. Another possible explanation for the clines detected in the present study is that they may be the result of selection acting on traits genetically correlated with body size. One candidate is developmental time, however there is no evidence for clinal latitudinal variation for this trait in the lines used in our work [81], in agreement with studies perfomed so far [20, 96]. On the contrary, starvation resistance exhibited a positive latitudinal cline in this set of lines [103]. This is interesting because one of the explanations proposed to explain the recurrently observed clinal patterns for body size is the “Starvation resistance hypothesis” [104–105]. This premise stands that larger body mass increases starvation resistance, which may be an advantage at high latitudes where resources are often seasonally scarce. Therefore, our observations not only give support to this idea but also suggest the existence of natural variation for genetic factors associated to body size and starvation resistance in *D*. *melanogaster* second chromosome.

Analyses testing the effects of altitude on morphological traits indicate a negative relationship, which is opposite to the altitudinal pattern observed in previous studies for different body size traits in *D*. *melanogaster* [29, 31, 34]. Sambucetti *et al*. [32] also found a negative association between thorax length and altitude in males of the cactophilic fly *D*. *buzzatii*. These results as well as ours are not consistent with the idea of thermal adaptation that has been recurrently invoked to explain body size variation along latitudinal and altitudinal gradients as a consequence of longer developmental times at lower temperatures [29, 31, 34]. However, there are other environmental factors that change in different manners along latitudinal and altitudinal gradients as thermal amplitude, which has been proposed as an explanation for patterns similar to those observed in our work [106–107]. In fact, these studies showed that stressful thermal regimes produced mean trait values smaller than those observed under constant conditions. Other environmental factors affecting insects’ performance that vary with altitude are air density and oxygen partial pressure, [37, 41]. In particular, it has been shown that flight performance might be strongly compromised by the combination of low temperature and low air density found at high altitudes [35]. In this respect, different morphological traits have been studied due to their effect on flight performance [35, 42–43]. Wing loading, a variable that combines wing and thorax size, is probably the most studied trait regarding flight performance in *Drosophila* and it has been generally proposed that low wing loading values may represent an adaptation to flight in cold environments [10, 18, 32, 34, 46–48, 108–109]. However, our results show that wing loading increases with latitude in both sexes. Even though this observation is contrary to the adaptive clinal variation hypothesis, similar results have been observed in South American populations of *D*. *subobscura* [44]. As the authors of the latter work suggested, factors other than flight may have favored the evolution of large overall size at low environmental temperatures. On the other side, wing loading decreased with altitude as expected considering that a large wing loading might severely compromise flight performance given the specific environmental conditions found at high altitudes (i.e. low temperature and air density; [41]). Even though reductions in wing loading could be achieved either through a reduction in body size or an increment in wing size; reducing body mass may be more expensive than enlarging wing size in terms of fitness, as it may imply a significant reproductive cost, especially for females [34, 110–114]. In fact, wing loading decreased with altitude as a consequence of a larger decrement in thorax length with respect to wing size. Moreover, this effect was more pronounced in males than in females, which is the sex expected to be more constrained in terms of reproductive costs.

Finally, wing morphology might also affect flight capacity as suggested by studies of wing aspect indicating that larger values of this composite trait are commonly associated to lower temperatures in *D*. *melanogaster* [20, 48–49]. In this respect, our results show that wings tend to be more elongated in populations at higher latitudes and more rounded at lower latitudes in both sexes. Besides, we observed a similar displacement of the posterior crossvein in both sexes, which tended to lean towards the proximal part of the wing as latitude increased. These changes are consistent with those observed in previous studies of wing aspect variation in natural populations of *D*. *melanogaster* [20, 48–49, 52]. Furthermore, other investigations revealed similar patterns for wing shape changes in *D*. *subosbscura* along latitudinal gradients [22]. All in all, these observations indicate that the posterior and distal portion of the wing is less functionally constrained than the anterior and proximal one, as suggested by previous work on insects flight [115]. Moreover, as vein patterning is much conserved among *Drosophila* species, especially in the highly derived *D*. *melanogaster* [68]; the similarity of the observations along different latitudinal gradients might imply certain degree of canalization of wing shape.

In spite of the similarity of the observed patterns with respect to previous observations, it must be stated that signals for clinality were very weak in our study. In fact, our analyses showed that the model considering only the population factor was always better than the others in explaining natural variation for morphological traits. However, it is worth mentioning that most of the populations were significantly different from the others at least for one trait in one sex. Furthermore, the most divergent populations were Neuquén, the southernmost locality sampled in the present study; Uspallata, the highest locality sampled; and Jáchal, a mid-low latitude and mid-high altitude location. These results suggest that the population factor involved more information than latitude and altitude, either individually or in combination. Moreover, in our case, this factor seems to include information regarding these gradients simultaneously with genetic aspects that might be differentiating populations, which must be associated to the second chromosome, as the lines are otherwise genetically identical.

Finally, quantitative genetic analyses showed that intra-population variation due to genetic factors (variance among lines and variance among lines dependent on sex) accounted for up to 41% of overall phenotypic variance, indicating significant within-population genetic variation which must be explained by factors present in the second chromosome as the lines are otherwise genetically identical. In fact, ~40% is the maximum value expected by the size of the respective genomic region. These results are in line with previous studies that found important genetic variation for morphological traits in *D*. *melanogaster* worldwide [7, 16, 20, 48, 49–52]. In particular, they are partially consistent with work showing a significant contribution of the second chromosome to variation for different body size related traits [21, 53–54, 56]. For example, mapping analyses showed that the right arm of the second chromosome was associated with QTL for body size traits in different continents and that this region was pleiotropic or contained closely linked QTL with predominant effects on only one trait [21, 54]. Other type of study found significant genetic variation for body size related traits spread across megabases surrounding the second chromosome centromere [56]. Regarding wing shape, Mezey *et al*. [55] found many QTLs in the second chromosome although most of them were different from those identified in previous studies [57–59]. In fact, seven out of eight QTL located in the second chromosome were specific of their study [55].

### Natural variants of genes related to morphological traits

Quantitative complementation tests indicated failure of complementation for all candidate genes tested as the interaction between the mutant background and the wild-derived strains was significant for a minimum of two traits in each case [73, 92–93]. These results support the hypothesis that natural variation at those loci affects the studied characters, thus suggesting that they are Quantitative Trait Genes (QTGs) for morphological traits [73, 92–93]. In particular, two genes showed natural genetic variation for face width (*Fasciclin 3* and *Reticulon-like1*) and wing shape (*jing* and *Reticulon-like1*), five genes for thorax length (*CG14478*, *Fasciclin 3*, *invected*, *Reticulon-like1* and *toucan*) and wing size (*CG14478*, *Fasciclin 3*, *invected*, *jing* and *Reticulon-like1*) and the six genes for head width. *CG14478*, *jing* and *Reticulon-like1* were the only genes affecting a single trait in a sex-specific manner, implying that genetic variation generally affects both sexes similarly. These analyses indicate that standing variation for the studied genes affecting morphological traits might be even higher than that registered in previous efforts regarding other quantitative traits in *D*. *melanogaster* [76–77, 79, 81, 116]. This is interesting because we employed co-isogenic chromosome substitution lines which also share the genetic background with the mutant lines used, which might have reduced the level of genetic variation in relation to previous studies that employed chromosomes carrying deficiencies and different genetic backgrounds [72–73, 92]. On the other hand, we used a larger number of alleles (i.e. second chromosome substitution lines) in comparison to previous works, which might have increased the amount of genetic variation detected.

Larger differences between wild type alleles in the mutant background with respect to the control background would suggest allelism as the predominant genetic mechanism generating phenotypic variation among lines [73, 92–93]. Certainly, a greater variance over the mutant background with respect to the control background would suggest allelism as the mechanisms generating such variation while the opposite pattern would suggest epistasis [81, 94]. Comparisons of genetic variances across mutant and control backgrounds showed that they were similar for all candidate genes suggesting that different natural alleles of each candidate gene might be responsible for a portion of the observed variation for morphological traits. However, non-additive effects due to interactions with other second chromosome loci affecting these traits cannot be ruled out, as wild-derived strains differ at myriads of loci in the second chromosome and any of these variants may interact epistatically with the mutant allele [93]. In fact, a closer look to the phenotypic means corresponding to the wild-derived backgrounds combined with each lab-derived background (i.e., mutant and control) revealed different outcomes. In particular, we found larger differences in the mutant background than in the control background as well as the opposite. Further, some of those differences were in the right direction (i.e., the means of descendants of big wild-derived lines were larger than those of descendants of small wild-derived strains) and others were not. Therefore, different patterns could be appreciated for different traits and candidate genes suggesting that both, allelism and epistasis, may be contributing to natural variation for body size traits.

Regarding morphological traits, there are, to our knowledge, only a couple of studies that analyzed the contribution of allelic variation to wing shape in *D*. *melanogaster* [55, 68]. These studies also used a geometric morphometric approach but they did not control for the genetic background. Moreover, Mezey *et al*. [55] and Palsson & Gibson [68] tested different alleles corresponding to 10 and 14 genes, respectively, known to affect vein patterning in *D*. *melanogaster*. Three of these genes were analyzed in both studies (*decapentaplegic*, *engrailed* and *rhomboid*) and none of them was tested in the present work, although *engrailed* is closely related to *invected* in genome position, sequence and pattern of expression [117]. Apart from these specific studies, the rest of the efforts regarding the analysis of natural genetic variation for morphological traits correspond to QTL mapping studies which have been already mentioned [21, 53–54, 57, 59]. In this regard, some of the QTLs identified for wing shape, wing area and thorax length are associated to the candidate genes tested in our study (Table 5). In particular, three different QTLs are related to *invected*, three to *jing* and three to *toucan*; two QTLs are associated to *CG14478* and two to *fasciclin 3* and, finally, one QTL is linked to *Reticulon-like1* (Table 5). Finally, Turner *et al*. [56] re-sequencing of populations selected for body size produced a list of genes that includes *CG14478*, *Reticulon-like1* and *toucan*.

**Table 5:** QTLs identified in previous studies associated to the candidate genes tested in this work.

This work		Previous studies					
Candidate Gene	Cytological position	Bergland *et al*. [53]^1^^,^^2^	Calboli *et al*. [21]^1^^,^^3^	Gockel *et al*. [54]^3^	Mezey *et al*. [55]^4^	Weber *et al*. [57]^4^	Zimmerman *et al*. [59]^4^
*toucan*	23D1-23D2	(21D1-23D1)(23A1-25D4)			Q5 (22C-24CD)		
*Reticulon-like1*	25B9-25C1					2 (25A3-27B1)	
*fasciclin 3*	36F2-36F4			3 (34D-44D)	Q6 (31F-36F)		
*Jing*	42B1-42B2			3 (34D-44D)	Q7 (41F-42B)Q8 (42B-47E)		
*invected*	47F15-47F17			4 (44A-52D)	Q9 (47E-48A)		B3.2 (47CF)
*CG14478*	54B16		(53E-56B)	5 (52B-56B)			

The names of the QTLs identified in previous studies are given as in the original publications when they are available. The cytological positions of the QTLs are indicated between parentheses.

^1^ QTLs for thorax length.

^2^ QTLs involved in epistatic interactions.

^3^ QTLs for wing area.

^4^ QTLs for wing shape.

Recent whole-genome analyses of *D*. *melanogaster* populations have revealed numerous clinally varying genes, which are shared across continents suggesting that many polymorphic sites are targets of natural selection [118–122]. Even though such genetic variation has rarely been connected to variation in fitness related traits or to causal clinal selection pressures, authors have speculated on its adaptive function by combining phenotypic and genomic data [122]. For example, it has been recognized a parallel differentiation between continents in genes associated with *D*. *melanogaster* wing morphogenesis [120], which might be related to the well-known wing size clines [122]. In spite of this, most of the mentioned reports have apparently not identified any of our six genes except for the study of Fabian *et al*. [118], which included *invected*, *Fasciclin 3* and *toucan* in the list of top candidate genes.

The present study as well as our previous investigations aimed to identify candidate genes for morphological traits [67, 75] showed that *invected* is a highly pleiotropic gene. Moreover, we have also shown that *invected* is a candidate gene for developmental time [123] and presents variants affecting this trait in the natural populations studied in this work [81]. As it was mentioned before, *invected* and *engrailed* are closely related genes [117]. Both are functionally redundant transcription factors that have been associated to the anterior-posterior patterning in the *Drosophila* wing [124]. The early expression of these genes during embryo development [125] might explain the general effect of *invected* on different body size related traits. Similarly, *jing* is a pleiotropic gene affecting several morphological traits [67, 75] and, also, a candidate gene for developmental time [123]. *jing* is a zinc finger like transcription factor [126–127] required for wing development and to establish the proximal-distal axis of the leg in *D*. *melanogaster* [126]. It has also been related to the process of regeneration [128] and the developing CNS midline and trachea [129]. Therefore, our results are in agreement with the multiple biological processes assigned to *jing*, mostly related to post-embryonic development and organ morphogenesis.

Regarding *Fasciclin 3* our results revealed that it is also a highly pleiotropic gene, though previous studies showed that it affects a small number of traits [67, 75, 123]. This gene encodes an immunoglobulin-like cell adhesion molecule which has been recently related to the control of tissue morphology mediated by intercellular adhesion [130], which is consistent with our observations. Likewise, complementation tests showed that *Reticulon-like1* is a highly pleiotropic gene in relation to morphological traits, although previous observations indicated a low level of pleiotropy [67, 75, 123]. Among the few biological processes related to this gene is aggressive behaviour in *D*. *melanogaster* [131], which usually include head interactions [132] that might be affected by head size (i.e. face and head width). On the contrary, this work showed that variation in *toucan* affected a few morphological traits although previous observations indicated that this is a highly pleiotropic gene [67, 75, 123]. In spite of this, we have not detected a single reference related to organ development or tissue morphogenesis that may be linked to our results. Finally, these as well as our previous results indicate that *CG14478* is a pleiotropic gene affecting different morphological traits as well as developmental time [67, 74–75, 123]. In fact, there are no more references linking this gene to any biological processes except for the mentioned work of Edwards *et al*. [131] that has associated it to aggressive behaviour in *D*. *melanogaster*.

In conclusion, the candidate genes tested are pleiotropic genes that exhibit high levels of variation (i.e. different alleles with dissimilar phenotypic effects) for morphological traits in natural populations of *D*. *melanogaster*. More specific studies are necessary to elucidate their participation during fly development and the molecular and functional basis of the natural variation detected as well as the processes acting upon it in nature.

## Supporting Information

S1 Appendix Example of the scripts used to perform analyses in R. The complete script used to analyze FW in females. Similar scripts were used to analyze the rest of the traits in females and all the characters in males.
(PDF)

S2 Appendix Head and thorax raw data corresponding to the substitution lines. Face width, head width and thorax length measurements corresponding to the studied individuals of the second chromosome substitution lines.
(XLSX)

S3 Appendix Wing size raw data corresponding to the substitution lines. Log_10_(Centroid size) values corresponding to the left wing of the studied individuals of the second chromosome substitution lines.
(XLSX)

S4 Appendix Wing shape raw data corresponding to the substitution lines. Log_10_(Procrustes distances) corresponding to the left wing of the studied individuals of the second chromosome substitution lines.
(XLSX)

S5 Appendix Head and thorax raw data corresponding to complementation tests for *invected*. Face width, head width and thorax length measurements corresponding to the studied individuals derived from the complementation tests performed for *invected*.
(XLSX)

S6 Appendix Wing size and shape raw data corresponding to complementation tests for *invected*. Log_10_(Centroid size) values and Log_10_(Procrustes distances) corresponding to the left wing of the studied individuals derived from the complementation tests performed for *invected*.
(XLSX)

S7 Appendix Head and thorax raw data corresponding to complementation tests for *Fasciclin 3*. Face width, head width and thorax length measurements corresponding to the studied individuals derived from the complementation tests performed for *Fasciclin 3*.
(XLSX)

S8 Appendix Wing size and shape raw data corresponding to complementation tests for *Fasciclin 3*. Log_10_(Centroid size) values and Log_10_(Procrustes distances) corresponding to the left wing of the studied individuals derived from the complementation tests performed for *Fasciclin 3*.
(XLSX)

S9 Appendix Head and thorax raw data corresponding to complementation tests for *toucan*. Face width, head width and thorax length measurements corresponding to the studied individuals derived from the complementation tests performed for *toucan*.
(XLSX)

S10 Appendix Wing size and shape raw data corresponding to complementation tests for *toucan*. Log_10_(Centroid size) values and Log_10_(Procrustes distances) corresponding to the left wing of the studied individuals derived from the complementation tests performed for *toucan*.
(XLSX)

S11 Appendix Head and thorax raw data corresponding to complementation tests for *Reticulon-like1*. Face width, head width and thorax length measurements corresponding to the studied individuals derived from the complementation tests performed for *Reticulon-like1*.
(XLSX)

S12 Appendix Wing size and shape raw data corresponding to complementation tests for *Reticulon-like1*. Log_10_(Centroid size) values and Log_10_(Procrustes distances) corresponding to the left wing of the studied individuals derived from the complementation tests performed for *Reticulon-like1*.
(XLSX)

S13 Appendix Head and thorax raw data corresponding to complementation tests for *jing*. Face width, head width and thorax length measurements corresponding to the studied individuals derived from the complementation tests performed for *jing*.
(XLSX)

S14 Appendix Wing size and shape raw data corresponding to complementation tests for *jing*. Log_10_(Centroid size) values and Log_10_(Procrustes distances) corresponding to the left wing of the studied individuals derived from the complementation tests performed for *jing*.
(XLSX)

S15 Appendix Head and thorax raw data corresponding to complementation tests for *CG14478*. Face width, head width and thorax length measurements corresponding to the studied individuals derived from the complementation tests performed for *CG14478*.
(XLSX)

S16 Appendix Wing size and shape raw data corresponding to complementation tests for *CG14478*. Log_10_(Centroid size) values and Log_10_(Procrustes distances) corresponding to the left wing of the studied individuals derived from the complementation tests performed for *CG14478*.
(XLSX)

S1 Fig Crosses performed to obtain second chromosome substitution lines. A single second chromosome was extracted from each isofemale line and substituted into the genetic background of an isogenic *Canton-S B* strain (IsoB) using balancer chromosomes carrying the following dominant phenotypic markers: *Curly* (*Cy*), *Stubble* (*Sb*), *Sternopleural* (*Sp*) and *white* eyes (*w*).
(JPG)

S2 Fig Head and thorax of a fly and related morphological traits. Picture showing the positioning of 3D body structures on a slide and related measurements taken with tpsDig.
(BMP)

S3 Fig Landmarks positioning on the ventral view of the left wing of a fly. LV: longitudinal vein, HCV: humeral cross vein, ACV: anterior cross-vein, PCV: posterior cross-vein.
(BMP)

S4 Fig Body size related traits with respect to geographical gradients. Mean values of Face Width (FW, blue), Head Width (HW, yellow), Thorax Length (TL, green) and Wing Size (WSi, red) in females (squares) and males (circles) of second chromosome substitution lines with respect to latitude (above) and altitude (below) of the natural population of origin. Bars indicate standard errors. FW, HW and TL values are in number of pixels. WSi is Centroid Size value x 10^-13^.
(TIFF)

S5 Fig Wing shape changes associated with latitude in males and females. Arrows indicate the magnitude and direction of landmark displacement with respect to the corresponding consensus wing shape (mean of the respective sex; females in red, males in blue). Arrow size has been magnified ten times to show wing shape changes more clearly. Circles indicate the largest landmark displacements. Vector diagrams were obtained using tpsRegr (http://morph.bio.sunysb.edu/morph/index.html).
(TIFF)

S6 Fig Second chromosome substitution lines ordered according to their size. Substitution lines were ordered according to the values of a composite score, which represent a general body size value estimated with the values corresponding to the four body size traits studied (face width, head width, thorax length and wing size; see text for details) in males (blue) and females (red). The six lines selected for the complementation tests are indicated.
(TIFF)

S1 Table Natural populations studied. Geographic information regarding sampling localities and number of second chromosome substitution lines derived from each one of them.
(PDF)

S2 Table Mutant lines used in Quantitative Complementation Tests. Information regarding the mutants, including the location of the mutations and their phenotypic effects.
(PDF)

S3 Table Genetic correlation analyses between morphological traits. Principal results of correlation analyses (r and R^2^ values) between body size related traits.
(PDF)

S4 Table Models testing the effect of latitude and altitude on morphological traits. Principal results of the models incorporating latitude and altitude independently and simultaneously to test the effect of these factors on the studied traits.
(PDF)

S5 Table Model selection. Principal results of model selection analyses based on the Akaike Information Criterion.
(PDF)

S6 Table Contrasts between populations for morphological traits. Principal results (p-values) of paired post-hoc Tukey tests between populations for each character in males and females separately. Bar: Barreal, Chi: Chilecito, Güe: Güemes, Jach: Jáchal, Lav: Lavalle, Nqn: Neuquén, Sbl: San Blas, Sj: San Juan, Usp: Uspallata. Significant values are shown in red.
(PDF)

S7 Table Correlation analyses between scores representing morphological traits. Principal results of correlation analyses (r and R^2^ values) between scores representing body size related traits.
(PDF)

S8 Table Descriptive statistics of morphological traits. Mean values and standard errors (SE) are shown per population for males and females separately.
(PDF)
