# Supplementary material for: Natural Genetic Variation and Candidate Genes for Morphological Traits in Drosophila melanogaster
Source: PLoS One. 2016 Jul 26;11(7):e0160069. doi: 10.1371/journal.pone.0160069 (PMC4961385; doi:10.1371/journal.pone.0160069)
Supplement: S1 Appendix — The complete script used to analyze FW in females. Similar scripts were used to analyze the rest of the traits in females and all the characters in males. (PDF) [file pone.0160069.s001.pdf]

```
FW_Females <-read.csv("FW_Females.csv", header=T)

library(car)

fit1<-lm(FW~Latitude,data=FW_Females)

summary(fit1)

Anova(fit1)

fit2<-lm(FW~Altitude,data=FW_Females)

summary(fit2)

Anova(fit2)

Fit3<-lm(FW~Altitude*Latitude,data=FW_Females)

summary(fit3)

Anova(fit3)

Fit4<-lm(FW~Population,data=FW_Females)

summary(fit4)

Anova(fit4)

AIC(fit1,fit2,fit3,fit4)

library(AICcmodavg)

Cand.mod <- list()

Cand.mod[[1]] <- fit1

Cand.mod[[2]] <- fit2

Cand.mod[[3]] <- fit3

Cand.mod[[4]] <- fit4

ModelNames <- c("Latitude", "Altitude", "Altitude*Latitude", "Population")

aictab(cand.set = Cand.mod, modnames = ModelNames, sort=T)

TukeyHSD(aov(FW~Population, data=FWFemales))
```
