# Supplementary figures and images for: Natural Genetic Variation and Candidate Genes for Morphological Traits in Drosophila melanogaster
Source: PLoS One. 2016 Jul 26;11(7):e0160069. doi: 10.1371/journal.pone.0160069 (PMC4961385; doi:10.1371/journal.pone.0160069)

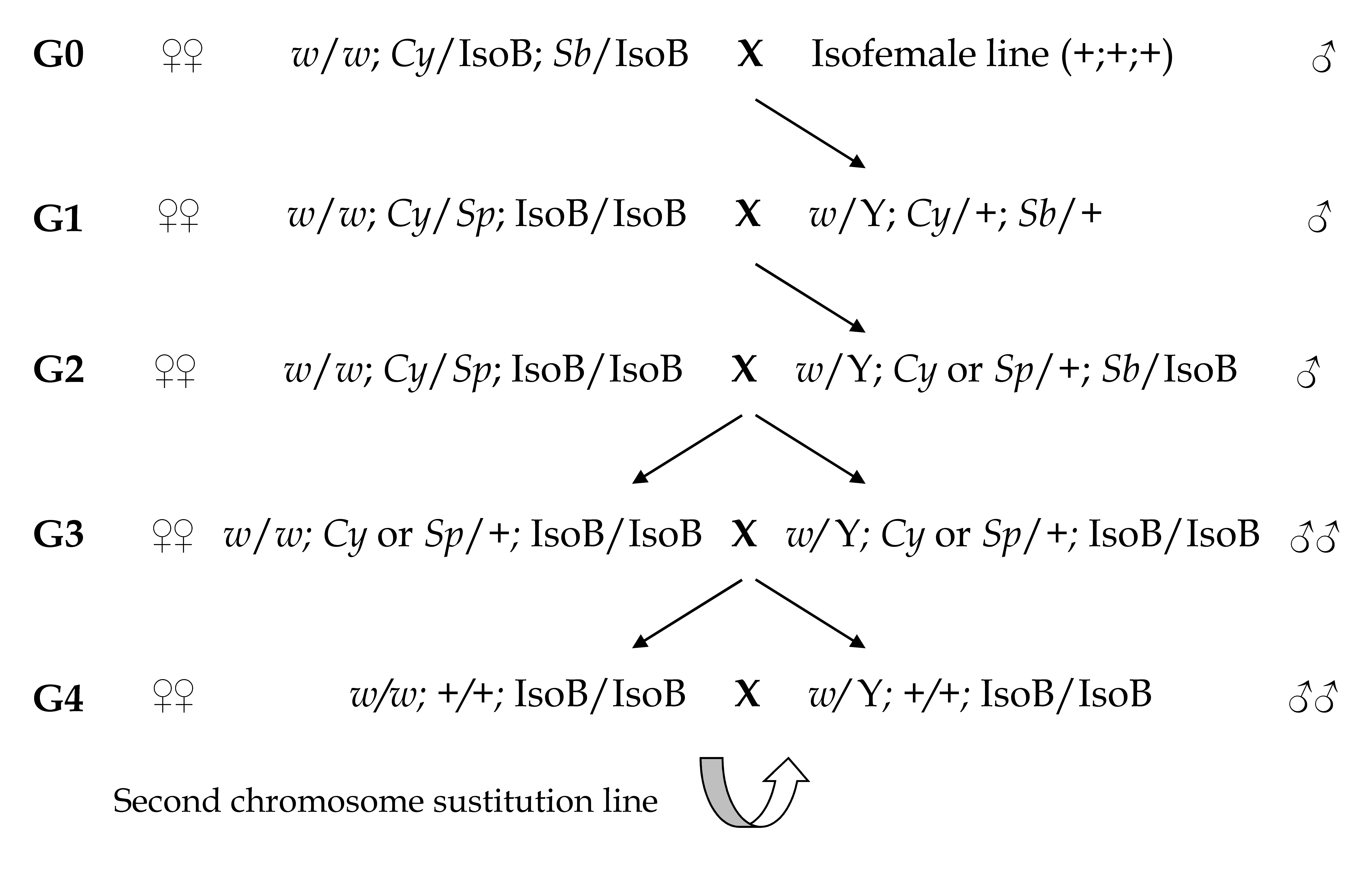

Supplement: S1 Fig — A single second chromosome was extracted from each isofemale line and substituted into the genetic background of an isogenic Canton-S B strain (IsoB) using balancer chromosomes carrying the following dominant phenotypic markers: Curly (Cy), Stubble (Sb), Sternopleural (Sp) and white eyes (w). (JPG) [file pone.0160069.s017.jpg]

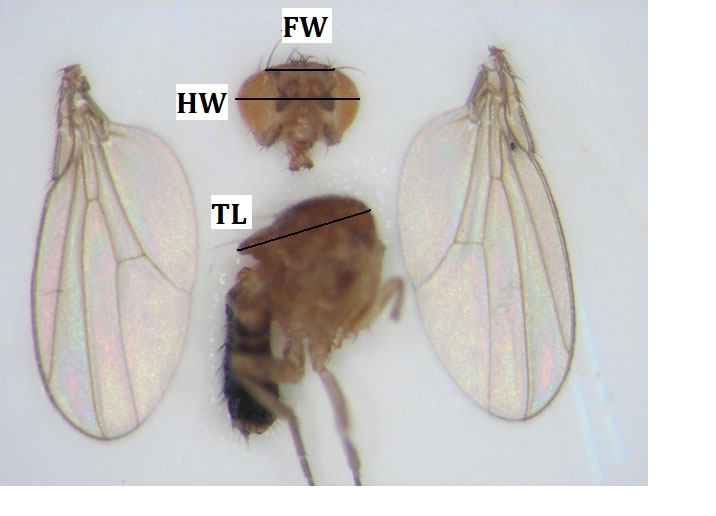

Supplement: S2 Fig — Picture showing the positioning of 3D body structures on a slide and related measurements taken with tpsDig. (BMP) [file pone.0160069.s018.bmp]

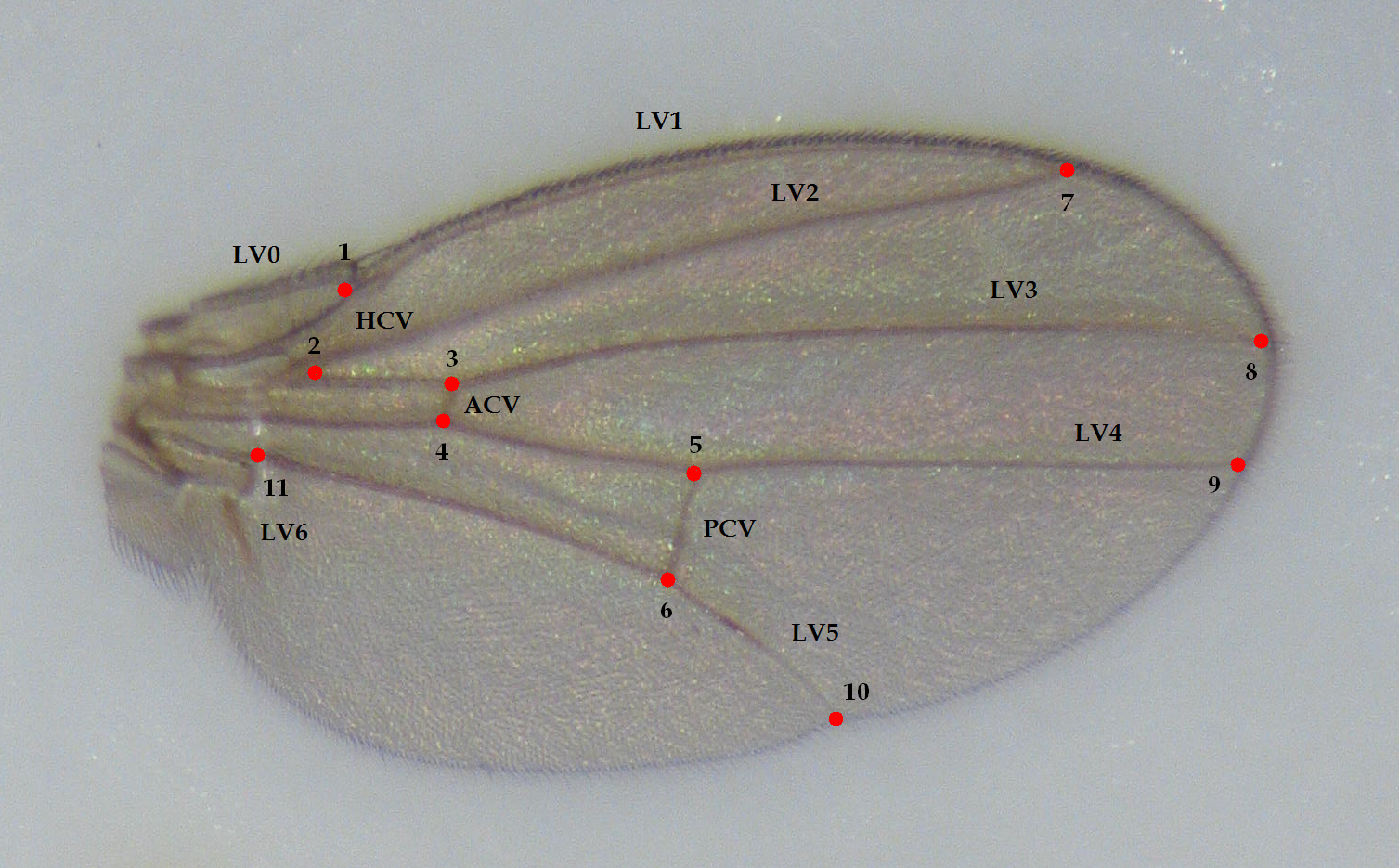

Supplement: S3 Fig — LV: longitudinal vein, HCV: humeral cross vein, ACV: anterior cross-vein, PCV: posterior cross-vein. (BMP) [file pone.0160069.s019.bmp]

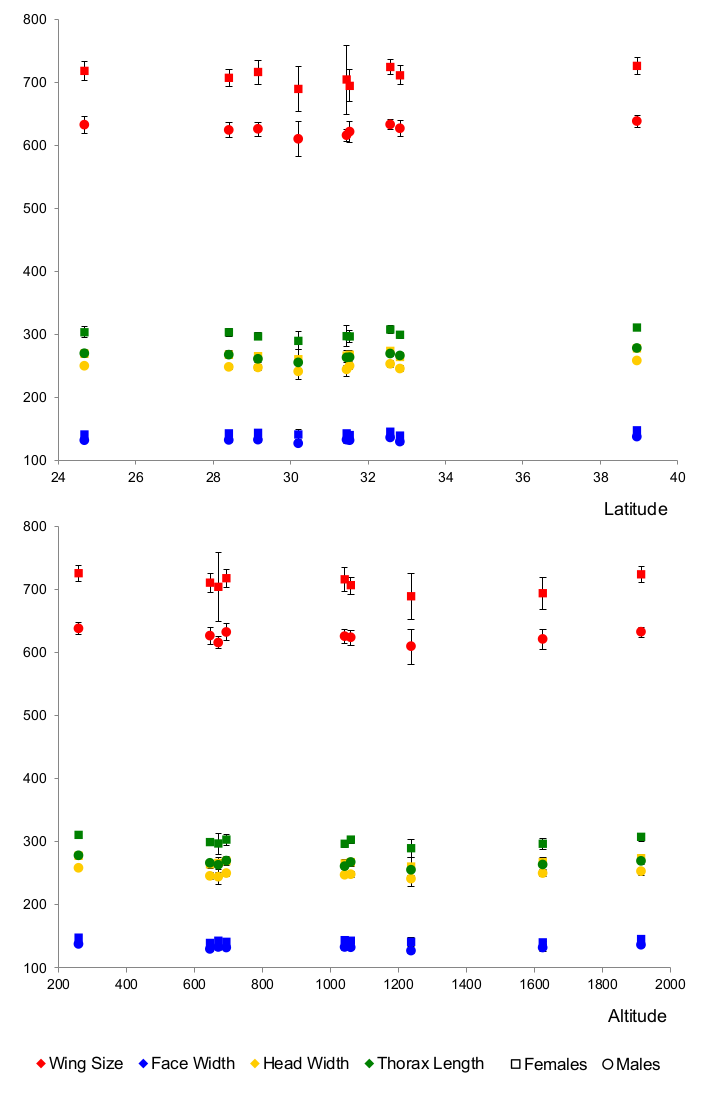

Supplement: S4 Fig — Mean values of Face Width (FW, blue), Head Width (HW, yellow), Thorax Length (TL, green) and Wing Size (WSi, red) in females (squares) and males (circles) of second chromosome substitution lines with respect to latitude (above) and altitude (below) of the natural population of origin. Bars indicate standard errors. FW, HW and TL values are in number of pixels. WSi is Centroid Size value x 10-13. (TIFF) [file pone.0160069.s020.tiff]

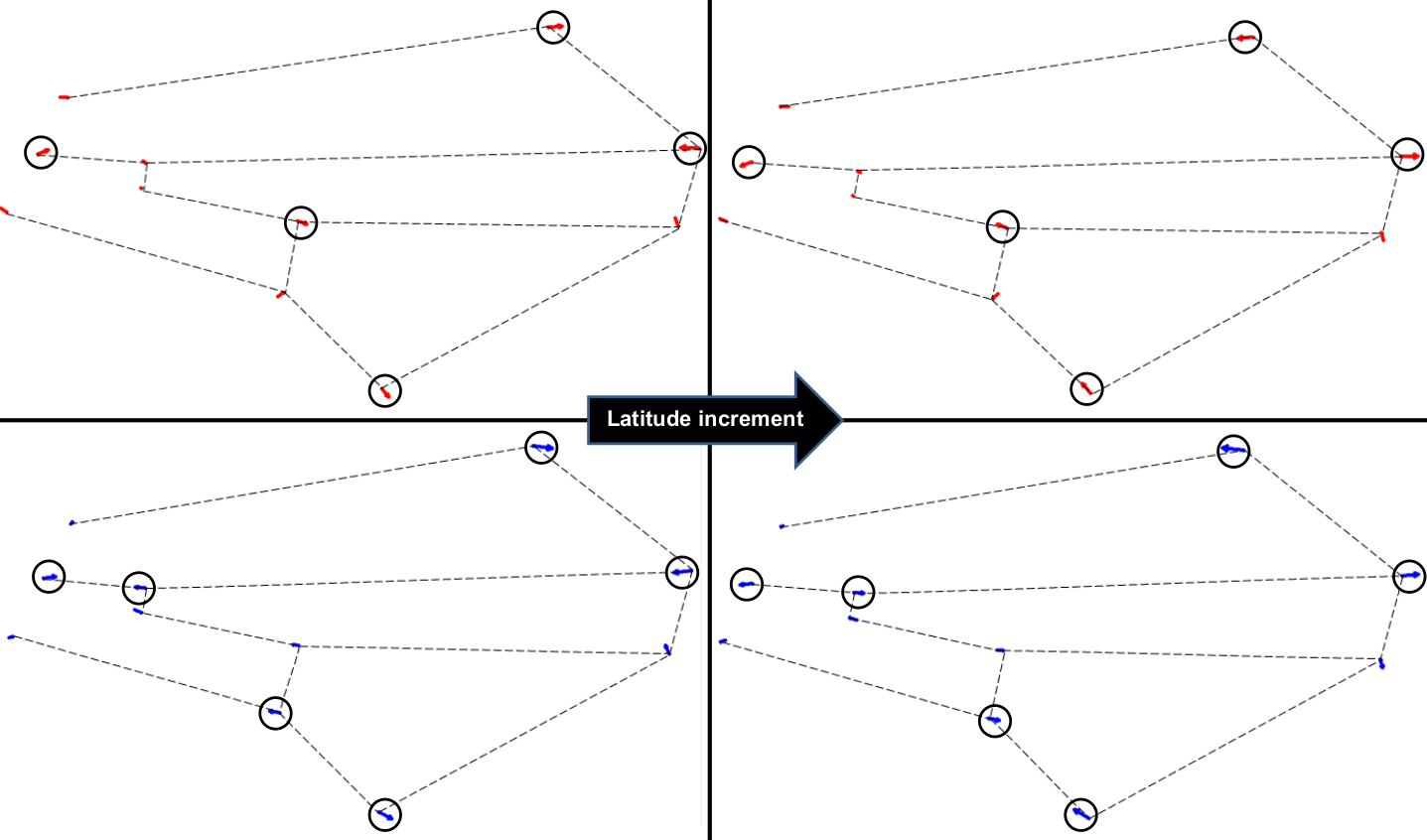

Supplement: S5 Fig — Arrows indicate the magnitude and direction of landmark displacement with respect to the corresponding consensus wing shape (mean of the respective sex; females in red, males in blue). Arrow size has been magnified ten times to show wing shape changes more clearly. Circles indicate the largest landmark displacements. Vector diagrams were obtained using tpsRegr (http://morph.bio.sunysb.edu/morph/index.html). (TIFF) [file pone.0160069.s021.tiff]

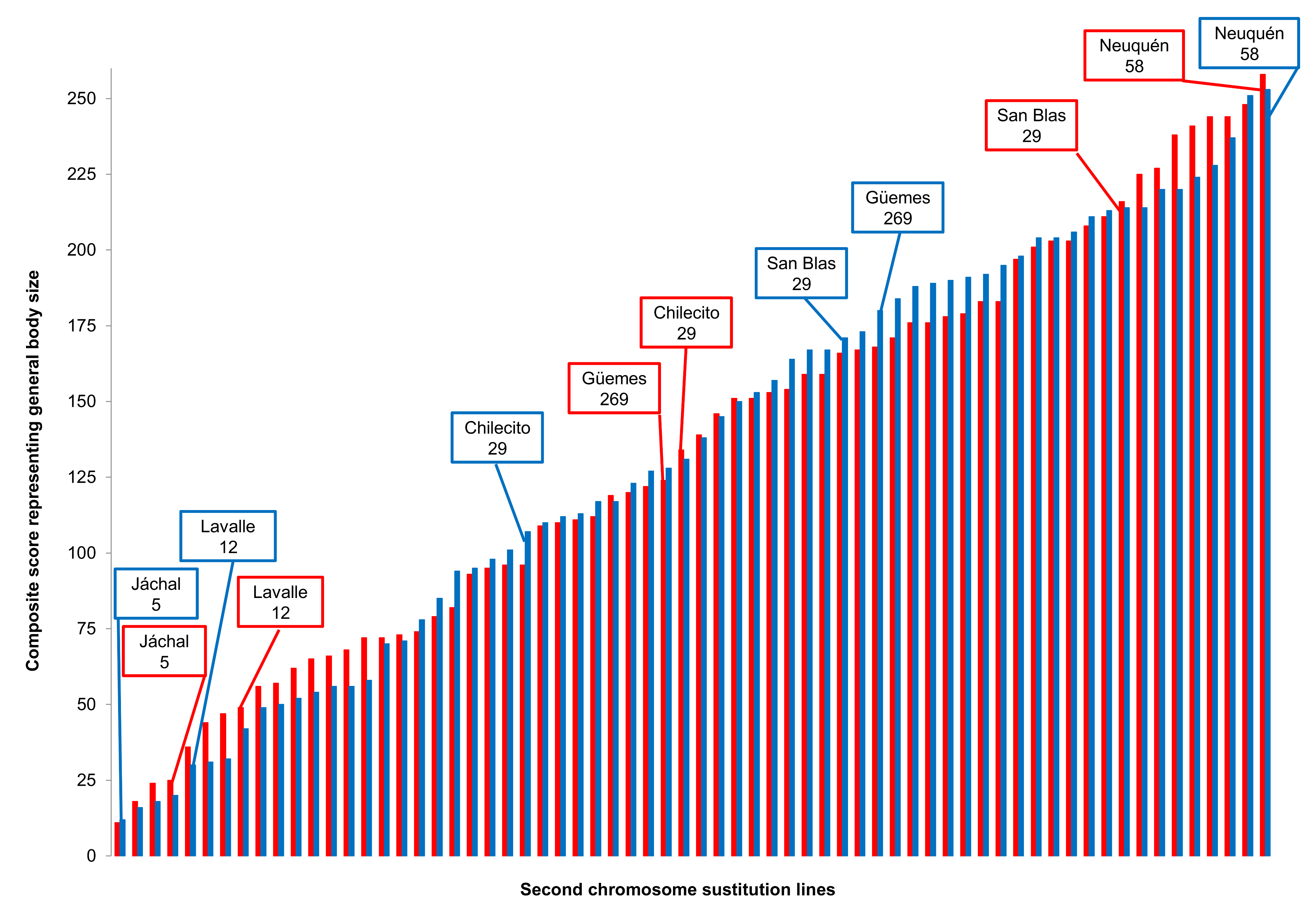

Supplement: S6 Fig — Substitution lines were ordered according to the values of a composite score, which represent a general body size value estimated with the values corresponding to the four body size traits studied (face width, head width, thorax length and wing size; see text for details) in males (blue) and females (red). The six lines selected for the complementation tests are indicated. (TIFF) [file pone.0160069.s022.tiff]
