# Supplementary material for: Natural Genetic Variation and Candidate Genes for Morphological Traits in Drosophila melanogaster
Source: PLoS One. 2016 Jul 26;11(7):e0160069. doi: 10.1371/journal.pone.0160069 (PMC4961385; doi:10.1371/journal.pone.0160069)
Supplement: S1 Table — Geographic information regarding sampling localities and number of second chromosome substitution lines derived from each one of them. (PDF) [file pone.0160069.s023.pdf]

S1 Table. Natural populations studied.

Population	Latitude (South)	Longitude (West)	Altitude over sea level	Number of lines
Güemes	24º 41’	65º 03’	695	10
San Blas	28º 25’	67º 06’	1061	9
Chilecito	29º 10’	67º 28’	1043	13
Jáchal	30º 12’	68º 45’	1238	3
San Juan	31º 27’	68º 31’	671	3
Barreal	31º 32’	69º 27’	1626	4
Uspallata	32º 35’	69º 22’	1915	6
Lavalle	32º 50’	68º 28’	647	8
Neuquén	38º 58’	68º 08’	260	10

Geographic information regarding sampling localities and number of second chromosome substitution lines derived from each one of them.
