# Supplementary material for: Natural Genetic Variation and Candidate Genes for Morphological Traits in Drosophila melanogaster
Source: PLoS One. 2016 Jul 26;11(7):e0160069. doi: 10.1371/journal.pone.0160069 (PMC4961385; doi:10.1371/journal.pone.0160069)
Supplement: S2 Table — Information regarding the mutants, including the location of the mutations and their phenotypic effects. (PDF) [file pone.0160069.s024.pdf]

S2 Table. Mutant lines used in Quantitative Complementation Tests.

Mutant line	Candidate gene	*p[GT1]* insertion site	Face Width		Head Width		Thorax Lenght		Wing Size		Wing Shape	
			♂	♀	♂	♀	♂	♀	♂	♀	♂	♀
BG00846	*invected*	In gene	ns	▼**	▼***	▼***	▼***	▼***	▼***	▼***	***	***
BG02023	*Fasciclin 3*	In gene	▲**	ns	ns	ns	ns	ns	ns	▼*	ns	***
BG02065	*toucan*	In gene	ns	▼***	▲***	ns	▲***	▼***	ns	▲**	*	*
BG02081	*Reticulon like 1*	In gene	ns	ns	ns	ns	▲***	ns	▲***	ns	ns	ns
BG02314	*jing*	289 bp at 3' side	▲*	ns	▲***	▼**	▲***	▼*	ns	ns	ns	**
BG02690	*CG14478*	In gene	▲*	ns	ns	▼***	ns	ns	ns	▼***	***	***

The candidate gene affected by the *P*-element insertion as well as the location of the transposon are given for each mutant line. Also, the phenotypic effect of the mutation is shown for each one of the morphological traits studied in males and females separately, according to [67, 75]. ▼ and ▲ represent significant size decrements and increments with respect to the control respectively. ns: not significant, * $p < 0.05$, ** $p < 0.01$, *** $p < 0.001$.
