# Supplementary material for: Natural Genetic Variation and Candidate Genes for Morphological Traits in Drosophila melanogaster
Source: PLoS One. 2016 Jul 26;11(7):e0160069. doi: 10.1371/journal.pone.0160069 (PMC4961385; doi:10.1371/journal.pone.0160069)
Supplement: S3 Table — Principal results of correlation analyses (r and R2 values) between body size related traits. (PDF) [file pone.0160069.s025.pdf]

S3 Table: Principal results of genetic correlation analyses between body size traits.

	Males			
**Females**	Face width	Head width	Thorax length	Wing size
Face width	0.69 (0.47)	0.79 (0.62)	0.66 (0.43)	0.67 (0.45)
Head width	0.72 (0.52)	0.83 (0.69)	0.78 (0.60)	0.74 (0.54)
Thorax length	0.60 (0.36)	0.80 (0.64)	0.87 (0.76)	0.78 (0.60)
Wing size	0.51 (0.26)	0.53 (0.28)	0.73 (0.54)	0.77 (0.60)

r and R^2 values (between parentheses) corresponding to correlation analyses performed between each pair of variables within males (in blue, above the diagonal), within females (in red, below the diagonal) and between sexes for each variable (in black, on the diagonal). p≤0.00001 in all cases ($P_{Bonferroni}$=0.0125).
