# Supplementary material for: Natural Genetic Variation and Candidate Genes for Morphological Traits in Drosophila melanogaster
Source: PLoS One. 2016 Jul 26;11(7):e0160069. doi: 10.1371/journal.pone.0160069 (PMC4961385; doi:10.1371/journal.pone.0160069)
Supplement: S4 Table — Principal results of the models incorporating latitude and altitude independently and simultaneously to test the effect of these factors on the studied traits. (PDF) [file pone.0160069.s026.pdf]

S4 Table: Principal results of the models testing the effect of latitude and altitude on morphological traits.

	Females						Males					
	**Estimate**	***t* value**	**p-value**	**RSE**	**df**	**MRS**	**Estimate**	***t* value**	**p-value**	**RSE**	**df**	**MRS**
**Face Width**												
**Model 1**												
Intercept	132.36	67.64	< 2e-16	8.92	1167	0.026	122.19	55.52	< 2e-16	10.09	1165	0.020
Latitude	0.35	5.57	3.19e-08				0.35	4.93	9.57e-07			
**Model 2**												
Intercept	143.70	240.38	<2e-16	9.03	1167	0.001	133.5	199.33	<2e-16	10.19	1165	0.001
Altitude	-5.89e-04	1.05	0.295				-6.47e-04	-1.02	0.308			
**Model 3**												
Intercept	103.40	14.01	< 2e-16	8.86	1165	0.040	108.2	12.92	< 2e-16	10.09	1163	0.023
Altitude	4.68e-02	4.09	4.66e-05				0.02	1.71	0.087			
Latitude	1.17	5.58	3.08e-08				0.74	3.11	0.002			
Altitude*Latitude	-1.41e-03	-4.08	4.80e-05				-6.70e-04	-1.70	0.089			
**Head Width**												
**Model 1**												
Intercept	250.99	93.14	< 2e-16	12.28	1167	0.039	230.63	88.88	< 2e-16	11.90	1165	0.045
Latitude	0.59	6.84	1.32e-11				0.62	7.41	2.4e-13			
**Model 2**												
Intercept	271.00	327.60	<2e-16	12.49	1167	0.005	251.6	315.28	<2e-16	12.14	1165	0.006
Altitude	-1.83e-03	-2.35	0.019				-2.01e-03	-2.66	0.008			
**Model 3**												
Intercept	226.30	22.19	< 2e-16	12.25	1165	0.045	210.6	21.34	< 2e-16	11.88	1163	0.050
Altitude	0.04	2.60	0.009				0.03	2.21	0.028			
Latitude	1.31	4.51	7.18e-06				1.20	4.28	2.02e-05			
Altitude*Latitude	-1.27e-03	-2.65	0.008				-1.04e-03	-2.25	0.025			
**Thorax Length**												
**Model 1**												
Intercept	284.57	83.35	< 2e-16	15.56	1168	0.023	248.71	77.40	< 2e-16	14.76	1166	0.030
Latitude	0.57	5.22	2.1e-07				0.62	5.99	2.78e-09			

Continue

	Females						Males					
	**Estimate**	***t* value**	**p-value**	**RSE**	**df**	**MRS**	**Estimate**	***t* value**	**p-value**	**RSE**	**df**	**MRS**
**Thorax Length**												
**Model 2**												
Intercept	305.50	294.89	<2e-16	15.66	1168	0.010	273.20	282.46	<2e-16	14.74	1166	0.032
Altitude	-3.38e-03	-3.48	5.2e-04				-5.73e-03	-6.25	5.68e-10			
**Model 3**												
Intercept	276.90	21.42	< 2e-16	15.53	1166	0.028	258.40	21.28	< 2e-16	16.62	1164	0.049
Altitude	0.02	0.84	0.400				6.32e-03	-0.34	0.738			
Latitude	0.85	2.31	0.021				0.45	1.30	0.193			
Altitude*Latitude	-5.82e-04	-0.96	0.338				5.27e-05	0.09	0.926			
**Wing Loading**												
**Model 1**												
Intercept	16.97	78.09	< 2e-16	0.91	1158	0.020	14.87	78.09	< 2e-16	0.87	1156	0.027
Latitude	0.03	5.65	1.72e-06				0.03	5.65	2.07e-08			
**Model 2**												
Intercept	18.08	282.81	<2e-16	0.92	1158	0.020	16.24	282.81	<2e-16	0.03	1156	0.028
Altitude	-1.68e-04	-5.80	3.4e-03				-3.16e-04	-5.80	8.5e-09			
**Model 3**												
Intercept	16.62	21.79	< 2e-16	0.91	1156	0.023	15.29	21.24	< 2e-16	0.87	1154	0.043
Altitude	7.74e-04	0.65	0.514				-1.56e-04	-0.14	0.889			
Latitude	0.04	2.01	0.045				0.03	1.40	0.162			
Altitude*Latitude	-2.68e-05	-0.75	0.455				-2.89e-06	-0.09	0.932			
**Wing Size**												
**Model 1**												
Intercept	16.83	3865.24	< 2e-16	0.02	1164	0.016	16.78	4060.82	< 2e-16	0.02	1160	0.009
Latitude	6.01e-04	4.28	2.01e-05				4.27e-04	3.22	1e-03			
**Model 2**												
Intercept	16.86	12790.23	<2e-16	0.02	1164	0.009	16.80	13472.72	<2e-16	0.02	1160	0.010
Altitude	-4.08e-06	-3.29	1.02e-03				-4.04e-06	-3.41	6.79e-04			

Continue

	Females						Males					
	**Estimate**	***t* value**	**p-value**	**RSE**	**df**	**MRS**	**Estimate**	***t* value**	**p-value**	**RSE**	**df**	**MRS**
**Wing Size**												
**Model 3**												
Intercept	16.85	1019.25	< 2e-16	0.02	1162	0.021	16.80	1070.72	< 2e-16	0.02	1158	0.015
Altitude	-1.95e-05	-0.76	0.448				-2.42e-05	-0.99	0.321			
Latitude	2.34e-04	0.50	0.618				-3.66e-05	-0.08	0.934			
Altitude*Latitude	4.99e-07	0.64	0.520				6.36e-07	0.86	0.389			
**Wing Shape**												
**Model 1**												
Intercept	12.31	472.65	< 2e-16	0.12	1164	0.002	12.36	466.38	< 2e-16	0.12	1161	7.94e-05
Latitude	1.40e-03	1.67	0.096				-2.58e-04	-0.30	0.761			
**Model 2**												
Intercept	12.37	1573.20	<2e-16	0.12	1164	0.003	12.37	1548.31	<2e-16	0.12	1161	3.73e-03
Altitude	-1.40e-05	-1.89	0.059				-1.58e-05	-2.08	0.037			
**Model 3**												
Intercept	11.83	121.09	< 2e-16	0.12	1162	0.028	11.76	119.01	< 2e-16	0.12	1159	0.042
Altitude	8.10e-04	5.34	1.14e-07				1.02e-03	6.62	5.41e-11			
Latitude	0.02	5.55	3.49e-08				0.02	6.13	1.18e-09			
Altitude*Latitude	-2.49e-05	-5.42	7.22e-08				-3.13e-05	-6.75	2.39e-11			

Principal results of the models incorporating latitude and altitude independently [Model 1: lm (X~Latitude); Model 2: lm (X~Altitude), where X represents the character; see Materials and Methods for more details] and simultaneously [Model 3: lm (X~Latitude*Altitude)] to test the effect of these factors on morphological traits in males and females separately. Estimate: estimate of the intercept and the slope corresponding to the factor/s tested. RSE: Residual Standard Error, df: degrees of freedom, MRS: Multiple R-squared.
