# Supplementary material for: Natural Genetic Variation and Candidate Genes for Morphological Traits in Drosophila melanogaster
Source: PLoS One. 2016 Jul 26;11(7):e0160069. doi: 10.1371/journal.pone.0160069 (PMC4961385; doi:10.1371/journal.pone.0160069)
Supplement: S5 Table — Principal results of model selection analyses based on the Akaike Information Criterion. (PDF) [file pone.0160069.s027.pdf]

S5 Table. Model selection based on the Akaike Information Criterion.

Females			Character	Males		
**Model**	**K**	**AIC**		**Model**	**K**	**AIC**
**4**	10	8381.80	**Face width**	**4**	10	8663.74
**3**	5	8424.23		**1**	3	8711.89
**1**	3	8436.85		**3**	5	8712.94
**2**	3	8466.40		**2**	3	8734.92
**4**	10	9063.46	**Head width**	**4**	10	8998.11
**3**	5	9181.42		**3**	5	9094.60
**1**	3	9185.10		**1**	3	9096.20
**2**	3	9225.45		**2**	3	9142.91
**4**	10	9629.43	**Thorax Length**	**4**	10	9481.92
**3**	5	9744.19		**3**	5	9586.84
**1**	3	9746.66		**2**	3	9603.11
**2**	3	9761.60		**1**	3	9606.22
**4**	10	2982.36	**Wing Loading**	**4**	10	2862.66
**3**	5	3082.45		**3**	5	2958.86
**1**	3	3082.63		**2**	3	2973.00
**2**	3	3096.96		**1**	3	2974.73
**4**	10	-5925.51	**Wing size**	**4**	10	-5978.37
**3**	5	-5829.25		**3**	5	-5918.53
**1**	3	-5827.17		**2**	3	-5916.11
**2**	3	-5819.76		**1**	3	-5914.85
**4**	10	-1731.87	**Wing shape**	**4**	10	-1675.10
**3**	5	-1686.57		**3**	5	-1643.04
**2**	3	-1659.88		**2**	3	-1601.46
**1**	3	-1659.08		**1**	3	-1597.21

Model 1: lm (X~Latitude), Model 2: lm (X~Altitude), Model 3: lm (X~Latitude*Altitude), Model 4: lm (X~Population); where X represents the character. K: number of estimated parameters for the model. AIC: Akaike Information Criterion value for the model. The best model is that with the lowest value of AIC.
