# Supplementary material for: Natural Genetic Variation and Candidate Genes for Morphological Traits in Drosophila melanogaster
Source: PLoS One. 2016 Jul 26;11(7):e0160069. doi: 10.1371/journal.pone.0160069 (PMC4961385; doi:10.1371/journal.pone.0160069)
Supplement: S6 Table — Principal results (p-values) of paired post-hoc Tukey tests between populations for each character in males and females separately. Bar: Barreal, Chi: Chilecito, Güe: Güemes, Jach: Jáchal, Lav: Lavalle, Nqn: Neuquén, Sbl: San Blas, Sj: San Juan, Usp: Uspallata. Significant values are shown in red. (PDF) [file pone.0160069.s028.pdf]

	Females						Males					
**Comparison**	**FW**	**HW**	**TL**	**WL**	**WSi**	**WSh**	**FW**	**HW**	**TL**	**WL**	**WSi**	**WSh**
Nqn-Bar	0.0000000	0.0000000	0.0000000	0.0000000	0.0000000	0.0029232	0.0018625	0.0000256	0.0000000	0.0000000	0.0003667	0.0000108
Usp-Jach	0.0399527	0.0000000	0.0000000	0.0000000	0.0000000	0.0027344	0.0000002	0.0000000	0.0000000	0.0000001	0.0000013	0.0107133
Jach-Güe	1.0000000	0.0000046	0.0000000	0.0000003	0.0000037	0.0001671	0.0121275	0.0000099	0.0000000	0.0000000	0.0000001	0.0256059
Nqn-Chi	0.0044885	0.0000000	0.0000000	0.0000000	0.0002272	0.9999989	0.0015235	0.0000000	0.0000000	0.0000000	0.0000961	0.9998703
Nqn-Güe	0.0000000	0.0000000	0.0000271	0.0002451	0.0054363	0.0013468	0.0000282	0.0000000	0.0000590	0.0002753	0.5951246	0.1466157
Nqn-Jach	0.0000431	0.0000000	0.0000000	0.0000000	0.0000000	0.8322847	0.0000000	0.0000000	0.0000000	0.0000000	0.0000000	0.9108384
Nqn-Lav	0.0000000	0.0000000	0.0000000	0.0000000	0.0000007	0.9999986	0.0000000	0.0000000	0.0000000	0.0000000	0.0022306	0.0544202
Sbl-Nqn	0.0000264	0.0000000	0.0000087	0.0001559	0.0000000	0.2260484	0.0000260	0.0000000	0.0000000	0.0000000	0.0000004	0.0669324
Sj-Nqn	0.0068652	0.0000000	0.0000000	0.0000001	0.0000039	0.0277555	0.1760173	0.0000000	0.0000001	0.0000001	0.0000049	0.7905783
Jach-Chi	0.3497197	0.0005526	0.0022967	0.0198288	0.0000340	0.8964929	0.0005308	0.0037914	0.0060413	0.1295743	0.0009510	0.9829835
Sbl-Jach	0.9571076	0.0022994	0.0000001	0.0000025	0.1269710	0.9999964	0.0230280	0.0049463	0.0000001	0.0000081	0.0481222	0.9957780
Usp-Chi	0.8706581	0.0000943	0.0000017	0.0000016	0.0752650	0.0086223	0.2053661	0.0002950	0.0002907	0.0001136	0.3334677	0.0108675
Lav-Jach	0.8993758	0.6735818	0.0010271	0.0083148	0.0145849	0.7233658	0.7407545	0.2475812	0.0000069	0.0002993	0.0008175	0.0095819
Güe-Chi	0.0309366	0.8490956	0.0057126	0.0090787	0.9985682	0.0000859	0.9812804	0.5617677	0.0000009	0.0000003	0.1208017	0.0221901
Usp-Lav	0.0000005	0.0000000	0.0001858	0.0002190	0.0011061	0.1050798	0.0000054	0.0000010	0.4515879	0.4454795	0.6280802	1.0000000
Usp-Sbl	0.1622869	0.0000820	0.2307819	0.2227722	0.0000058	0.0000015	0.0167304	0.0010153	0.8795891	0.9105801	0.0215146	0.0000007
Jach-Bar	0.9954127	0.0135943	0.1361761	0.3661575	0.9999295	0.0002142	0.0717105	0.0000694	0.0048926	0.0460525	0.0787643	0.0000049
Usp-Sj	0.5063322	0.0004637	0.0002340	0.0004213	0.0008089	0.9992462	0.7427591	0.0014901	0.4186044	0.2330390	0.0091647	0.9976260
Güe-Bar	0.9682169	0.8814995	0.0054201	0.0104366	0.0000034	0.9986170	1.0000000	0.9999923	0.0143932	0.0190452	0.0801470	0.0573891
Usp-Bar	0.0002529	0.0017332	0.0000064	0.0000094	0.0000000	0.9841411	0.0901978	0.6223585	0.0896194	0.0981867	0.1796746	0.3749689
Chi-Bar	0.0077271	0.9999985	0.9918718	0.9946592	0.0000388	0.0005408	0.9906755	0.6189145	0.9977066	0.9864800	0.9953499	0.0000005
Lav-Chi	0.0000216	0.0352365	0.9997582	0.9995877	0.7726112	0.9997126	0.0217175	0.7588821	0.3133798	0.1948902	0.9999966	0.0068334
Sbl-Bar	0.2889289	1.0000000	0.0254090	0.0369930	0.2443919	0.0000001	1.0000000	0.7101235	0.6858617	0.6581229	0.9999997	0.0000000
Sbl-Güe	0.7922477	0.7403096	0.9999676	0.9999956	0.0161261	0.0000000	1.0000000	0.7000859	0.5088145	0.6145593	0.0026274	0.0000004
Usp-Nqn	0.6055419	0.0938899	0.3549227	0.7136273	0.9725470	0.0381091	0.9847903	0.0305507	0.0001828	0.0009339	0.7105192	0.0659972
Lav-Güe	0.5504784	0.0002253	0.1096052	0.1645100	0.3638071	0.0077845	0.3230793	0.0181193	0.1155518	0.1281544	0.3922244	0.9998111
Sbl-Chi	0.8710821	0.9999983	0.0444571	0.0535439	0.1001300	0.2786971	0.9584801	1.0000000	0.0289835	0.0092291	0.9088202	0.1738345
Sj-Jach	0.9861672	0.1478755	0.1190371	0.2776368	0.3985518	0.0019131	0.0109271	0.3363681	0.0031520	0.0618555	0.7654840	0.2521918
Usp-Güe	0.0008376	0.0237287	0.4094794	0.3289770	0.3419675	0.9999559	0.0211212	0.1500638	0.9999911	0.9999979	1.0000000	0.9991788
Lav-Bar	0.9998697	0.3616944	0.9298782	0.9365921	0.0292876	0.0100576	0.7165261	0.0519796	0.9727618	0.9777982	0.9805514	0.2302722
Sbl-Lav	0.0187193	0.1214119	0.3296890	0.3880451	0.9829163	0.1420463	0.4745771	0.7709161	0.9972062	0.9937496	0.8252727	0.0000001
Sj-Sbl	1.0000000	0.9980902	0.1420234	0.2100664	1.0000000	0.0000086	0.9875330	0.9899971	0.9687793	0.8408589	0.9753430	0.0048696
Sj-Chi	0.9796530	0.9872804	0.9998407	0.9999797	0.3812137	0.0090167	1.0000000	0.9916979	0.9639713	0.9837609	0.4705229	0.5228462
Sj-Güe	0.9597982	0.5364741	0.0520262	0.0988098	0.1471892	0.9999945	0.9943988	0.3789070	0.1735761	0.0802823	0.0024468	0.9999984
Sj-Bar	0.5984648	0.9994825	0.9999995	0.9999971	0.6191107	0.9999993	0.9947906	0.3794846	0.9999752	1.0000000	0.9652715	0.1660526
Sj-Lav	0.1944726	0.9080139	0.9925967	0.9964409	0.9915640	0.0653199	0.2227137	0.9999983	0.9998361	0.9953240	0.3822893	0.9990479
