# Supplementary material for: Natural Genetic Variation and Candidate Genes for Morphological Traits in Drosophila melanogaster
Source: PLoS One. 2016 Jul 26;11(7):e0160069. doi: 10.1371/journal.pone.0160069 (PMC4961385; doi:10.1371/journal.pone.0160069)
Supplement: S7 Table — Principal results of correlation analyses (r and R2 values) between scores representing body size related traits. (PDF) [file pone.0160069.s029.pdf]

S7 Table: Principal results of correlation analyses between scores representing body size traits.

	Males			
**Females**	Face width	Head width	Thorax length	Wing size
Face width	-	0.78	0.68	0.68
	-	(0.61)	(0.47)	(0.46)
Head width	0.75	-	0.78	0.73
	(0.56)	-	(0.61)	(0.53)
Thorax length	0.59	0.82	-	0.79
	(0.34)	(0.68)	-	(0.62)
Wing size	0.51	0.71	0.75	-
	(0.26)	(0.51)	(0.56)	-

r and R^2 values (between parentheses) corresponding to correlation analyses performed between each pair of variables represented by a morphological score (see Material and Methods for more details) in males (in blue, above the diagonal) and females (in red, below the diagonal) separately. p≤0.0001 in all cases ($P_{Bonferroni}$=0.0167).
