# Supplementary material for: Natural Genetic Variation and Candidate Genes for Morphological Traits in Drosophila melanogaster
Source: PLoS One. 2016 Jul 26;11(7):e0160069. doi: 10.1371/journal.pone.0160069 (PMC4961385; doi:10.1371/journal.pone.0160069)
Supplement: S8 Table — Mean values and standard errors (SE) are shown per population for males and females separately. (PDF) [file pone.0160069.s030.pdf]

S8 Table: Descriptive statistics of morphological traits.

	Face width		Head width		Thorax length		Log_{10} (Wing size)		Log_{10} (Wing shape)		N
	Mean	SE	Mean	SE	Mean	SE	Mean	SE ($x10^{-3}$)	Mean	SE ($x10^{-3}$)	
**Females**											
Güemes	141.30	0.66	269.41	0.89	303.75	1.25	16.86	1.85	12.32	7.70	190-191
San Blas	142.83	0.72	267.34	1.00	302.98	1.25	16.85	1.76	12.40	11.66	155-161
Chilecito	144.13	0.64	267.16	1.00	298.11	1.06	16.85	1.91	12.37	8.31	208-210
Jáchal	141.35	1.12	260.58	1.49	289.80	1.82	16.84	2.51	12.40	13.68	60
San Juan	142.92	1.10	266.29	1.49	297.29	1.96	16.85	2.83	12.31	12.39	61-62
Barreal	140.10	0.88	267.51	1.00	296.66	1.30	16.84	2.01	12.31	12.72	80-82
Uspallata	145.74	0.71	274.32	0.85	307.82	1.20	16.86	1.23	12.33	9.90	116-117
Lavalle	139.24	0.73	263.67	1.17	299.32	1.22	16.85	1.67	12.37	8.39	137-139
Neuquén	147.40	0.76	277.92	1.03	311.25	1.23	16.86	1.22	12.37	9.60	157-159
**Males**											
Güemes	132.17	0.86	249.99	0.92	270.71	1.13	16.80	1.31	12.33	7.83	181
San Blas	131.86	0.75	247.42	1.05	267.23	1.30	16.79	1.85	12.41	10.38	159-161
Chilecito	133.05	0.76	247.45	0.82	262.52	0.95	16.80	1.43	12.37	8.84	204-208
Jáchal	127.03	1.08	241.34	1.51	255.21	1.79	16.78	2.06	12.39	14.40	60-62
San Juan	132.65	1.15	244.39	1.70	263.07	2.19	16.79	2.73	12.35	16.12	60
Barreal	131.72	0.85	249.85	1.17	263.40	1.31	16.79	1.99	12.28	11.41	80-81
Uspallata	136.19	0.89	253.69	0.95	270.09	1.17	16.80	1.48	12.32	10.72	109-111
Lavalle	129.60	0.89	245.62	1.01	266.33	1.33	16.80	1.64	12.33	10.48	142
Neuquén	137.42	0.70	258.24	0.87	277.89	0.99	16.81	1.24	12.37	8.84	168-169

Mean values and standard errors (SE) are shown per population for males and females separately. N: sample size (different values indicate different sample sizes for different traits).
